# Discovery of druggable cancer-specific pathways with application in acute myeloid leukemia

**DOI:** 10.1093/gigascience/giac091

**Published:** 2022-09-29

**Authors:** Quang Thinh Trac, Tingyou Zhou, Yudi Pawitan, Trung Nghia Vu

**Affiliations:** Department of Medical Epidemiology and Biostatistics, Karolinska Institutet, Nobels väg 12A, Stockholm 17177, Sweden; School of Data Sciences, Zhejiang University of Finance and Economics, 310018 Hangzhou, China; Department of Medical Epidemiology and Biostatistics, Karolinska Institutet, Nobels väg 12A, Stockholm 17177, Sweden; Department of Medical Epidemiology and Biostatistics, Karolinska Institutet, Nobels väg 12A, Stockholm 17177, Sweden

**Keywords:** cancer-specific pathways, pathway activation score, AML

## Abstract

An individualized cancer therapy is ideally chosen to target the cancer’s driving biological pathways, but identifying such pathways is challenging because of their underlying heterogeneity and there is no guarantee that they are druggable. We hypothesize that a cancer with an activated druggable cancer-specific pathway (DCSP) is more likely to respond to the relevant drug. Here we develop and validate a systematic method to search for such DCSPs, by (i) introducing a pathway activation score (PAS) that integrates cancer-specific driver mutations and gene expression profile and drug-specific gene targets, (ii) applying the method to identify DCSPs from pan-cancer datasets, and (iii) analyzing the correlation between PAS and the response to relevant drugs. In total, 4,794 DCSPs from 23 different cancers have been discovered in the Genomics of Drug Sensitivity in Cancer database and validated in The Cancer Genome Atlas database. Supporting the hypothesis, for the DCSPs in acute myeloid leukemia, cancers with higher PASs are shown to have stronger drug response, and this is validated in the BeatAML cohort. All DCSPs are publicly available at https://www.meb.ki.se/shiny/truvu/DCSP/.

## Introduction

Cancer is the second leading cause of deaths and was responsible for 9.6 million deaths worldwide in 2018. Approximately 1 in 6 deaths is due to cancer [1]. Cancer can result from an uncontrollable cell growth due to genetic alterations in their genomes [2] that change the biological function of some oncogenes and their associated pathways. Drugs designed for specific gene targets may not work as expected in a specific cancer because of the underlying heterogeneity in its driving biological pathways. To kill a specific cancer with an inhibitor, theoretically we need to find one that can downregulate the cancer’s driving pathway(s). There are at least 2 immediate challenges: (i) pathway activation is only a necessary but not sufficient condition for its driving property, and empirically, we can observe many activated pathways in any given cancer, so it is not obvious how to determine which is the driving pathway, and (ii) the driving pathway may not have druggable targets, for example, the driving pathway has a poor functional connectivity with the targets of the drug, leading to no impact of the drug on the driving pathway. Thus, in our approach, a pathway activity is first measured by the mRNA expression of the genes in the pathway. The pathway activity is weighted by the functional connectivity between the pathway, potential driver genes, and drug targets. Then, we search for pathways that are uniquely activated in specific cancers but not in others. We focus on druggable pathways, roughly those have known drug targets. (In the actual computation, we also allow genes upstream to the targets.) We hypothesize that a cancer with an activated druggable cancer-specific pathway (DCSP) is more likely to respond to the relevant drug. Thus, our aim is to develop and validate a systematic method to search for such DCSPs.

Many studies [3–5] have investigated universal cancer signaling pathways. For instance, the p53, RTK–RAS signaling or cell cycle pathways are frequently altered across different cancers [6]. Recently, Sanchez and colleagues [7] analyzed the mechanisms and patterns of somatic alterations in 10 common canonical pathways in different cancers using The Cancer Genome Atlas (TCGA) cohort: cell cycle, Hippo, Myc, Notch, Nrf2, PI-3–Kinase/Akt, RTK–RAS, TGFβ signaling, p53, and β-catenin/Wnt. However, some altered signaling pathways appear limited to specific tumors; for example, some pathways of BRCA1 and BRCA2 tumor-suppressor genes are known to be specific to breast and ovarian cancers [8–10]. Altered signaling pathway due to the chromosomal rearrangement event of PML–RARA fusion [11] is often observed only in acute promyelocytic leukemia (APL), a distinct subgroup of acute myeloid leukemia (AML). Here we shall consider only pathways that are cancer specific.

For a given altered signaling pathway that is specific to a cancer, different drugs can affect the pathway differently, thereby potentially producing distinct levels of drug response. Conceptually, we expect the action of a drug from the role of its targets in the pathway. For instance, midostaurin and gilteritinib are inhibitors that target mutations of a type III receptor tyrosine kinase (FLT3) [12], which occur in 30% of AML cases [13]. So, the action of these inhibitors should be assessed in activated pathways that contain the FLT3 gene. Therefore, the investigation of a signaling pathway specific to a cancer is more informative clinically if it mediates the action of a specific drug. In other words, the pathway is druggable, so we need to capture the element of druggability in the definition of the pathway activity.

**Figure 1:**
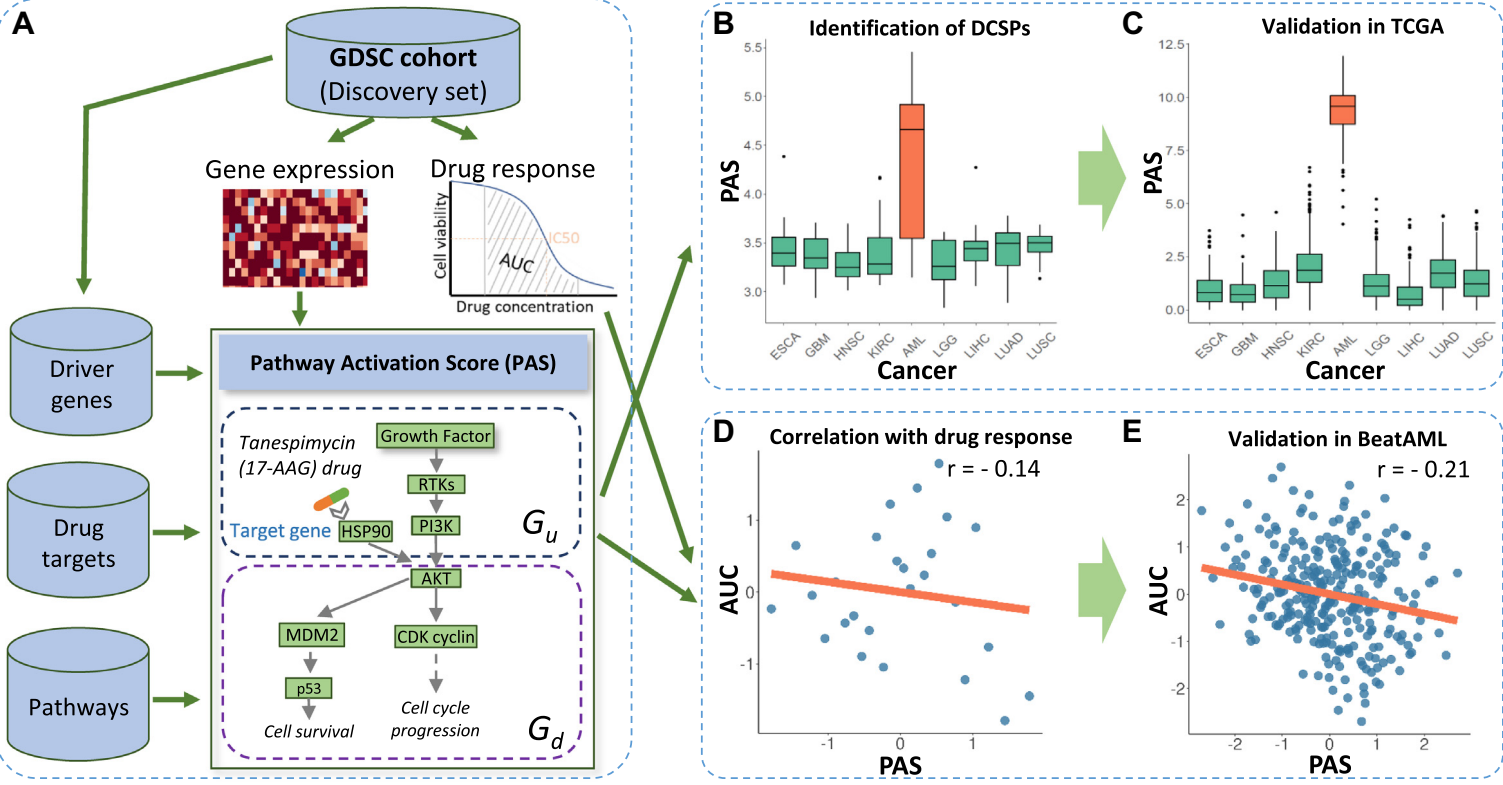
Overview of identifying DCSPs from the pharmacogenomics data. (A) PAS is computed from the pharmacogenomics data of the GDSC along with pathway and drug target databases. In the illustration of PAS, tanespimycin or 17-AAG has a target gene HSP90, which involves pathway PI3k/AKT. For simplicity, the full information of the pathway is not shown in this example. The main analysis includes (i) identification of DCSPs from the GDSC cohort with validation using the TCGA cohort (B, C) and (ii) investigation of the association between PAS and drug responses with validation using the BeatAML cohort (D, E). These plots are derived from the analyses of the PASs of Martens–PML–RARA [20] druggable by quizartinib in AML. The boxplots (B, C) show that the PAS of AML is overexpressed while the PASs of other cancers are low expressed and not significantly different from each other. (D, E) Each point presents a tumor, and the lines are linear regression lines. The values of PAS and area under the curve (AUC) in the plots are under the normal score transformation; see the Materials and Methods section.

Here we develop a systematic methodology to identify and validate druggable cancer-specific pathways. Briefly, we compute a pathway activation score (PAS) to represent the activity level of pathways for specific cancers and take drug targets into account. The PAS of a tumor is calculated for each drug–pathway pair using information of gene expression and driver genes of the tumor and target genes of the drug. Then, we implement cancer-specific analysis to discover the cancer-specific pathways (DCSPs) that exhibit high activation only in aingle cancer while activation scores of the pathways in other cancers are not significantly different from each other. The workflow of the study is presented in Fig. 1. First, we apply the proposed method to identify DCSPs from the Genomics of Drug Sensitivity in Cancer (GDSC) cohort [14] as the discovery set, which contains 23 different cancers and 251 drugs. Then, the DCSPs are validated in the TCGA cohort [15]. Finally, utilizing the fruitful omic and drug data of the BeatAML study [16], we focus on the DCSPs of AML, the most common type of leukemia cancer in adults with a high relapse rate (50% within 6 months) and poor survival outcome (only 10% within 5 years) [17, 18]. Some recent studies also consider the integration of the GDSC and the BeatAML cohort. For example, Jafari et al. [19] used the drug data from 2 cohorts to develop bipartite network models to search for potential combination therapies in AML. However, they did not focus on identifying potential biological pathways associated with the drug response. In support of our hypothesis, for the DCSPs in acute myeloid leukemia, cancers with higher PASs are shown to have stronger drug response, and this is validated in the BeatAML cohort.

## Results

### Pathway activation score

PAS is defined as a tumor-specific pathway activity level that is relevant to a specific drug. It is calculated based on the connection between the driver gene(s), the drug-specific target gene(s), and the tumor-specific mRNA expression level of the genes in the pathway. PAS of a tumor is calculated for each drug–pathway pair. Genes in a pathway *P* are classified into 2 groups: (i) *G*_*u*_, which includes both the target and upstream genes, and (ii) *G*_*d*_, which contains the downstream genes. We first compute an upstream activity score *S*(*G*_*u*_) as the sum of mRNA expression of the genes in *G*_*u*_. Next, the score is weighted by the functional network connectivity between the gene sets of the driver genes, the target genes, and the pathways using the network enrichment analysis (NEA) [21], which is described in further details in the Materials and Methods section. Three connectivity weights *w*_1_, *w*_2_, and *w*_3_ are computed for these pairs of gene sets: (driver genes ↔ target genes), (driver genes ↔ pathway gene sets), and (target genes ↔ pathway gene sets). Each weight ranges from 0 to 1, where 0 indicates little or no functional interaction and 1 indicates a high interaction. The final PAS_*u*_, the pathway score for upstream activity, is calculated as *S*(*G*_*u*_)*(1 + *w*_1_ + *w*_2_ + *w*_3_). In the implementation, we identify recurrent mutations and fusions in each tumor as the potential driver genes; more details are given in the Materials and Methods section. The pathway score for downstream activity PAS_*d*_ is computed similarly. Fig. 1A illustrates a toy example of PAS for PI3k/ATK pathway targeted by tanespimycin.

For the purpose of identifying DCSPs, we need to define a scalar PAS. Our hypothesis is that for a drug to be effective on a tumor, its target genes should be part of a pathway that is highly activated, where high activation is measured relative to the other part of the pathway. So we focus on the positive PAS ≡ PAS_*u*_ − PAS_*d*_ as the primary pathway activation score. The hypothesis will be further supported in terms of biological specificity if there is evidence that the downstream activation is not informative of drug response. As a motivation, suppose the driver is downstream of the target and that part of the pathway (PAS_*d*_) is highly activated, while the target (or PAS_*u*_) is not activated. This is the case where PAS_*d*_ − PAS_*u*_ > 0, where the downstream activation is measured relative to the upstream activation. So we also investigate this secondary version of PAS as a measure of version downstream activation and hypothesize that in this case, PAS does not correlate with drug response.

PAS is computed for a set of biological pathways *P* = *P*_1_,..., *P*_*N*_, a set of drugs *D* = *D*_1_,..., *D*_*M*_, and a set of tumor samples *S* = *S*_1_,..., *S*_*K*_ from *Z* types of cancers *C* = *C*_1_,..., *C*_*Z*_. A PAS of tumor sample *S*_*k*_, drug *D*_*i*_, and pathway *P*_*j*_ is PAS(*S*_*k*_, *D*_*i*_, *P*_*j*_), or simply PAS if it is clear from the context. Thus, given tumor *S*_*k*_, PAS is calculated for each (*D*_*i*_, *P*_*j*_) pair. In practice, we use *N* = 4,762 curated human pathways from the MSigDB database. Using the GDSC data as the discovery set, we have *M* = 251 drugs and *K* = 684 samples from *Z* = 23 cancer types. The target genes of drugs are provided from the GDSC cohort and extended with the curated information from the DrugBank database [22]. The direction of regulatory interactions between genes is taken from multiple directed network databases, including HTRIdb [23], regulatory target gene sets of the MSigDB database [24], transcriptional factor target database of the UCSC Genome Browser Database [25], and kinase–substrate interaction database [26].

### Identification of druggable cancer-specific pathways

Fig. 1 presents an overview of the process to identify DCSPs. First, the gene expression data from GDSC are obtained to calculate PAS. The list of the cancers, their abbreviation, and number of samples of each cancer are provided in Supplementary Table S1. Next, DCSP analysis is applied to discover DCSPs based on PASs. The DCSP analysis takes into account all drug–pathway–cancer triplets to discover highly activated druggable pathways that are specific to each cancer. Finally, the DCSPs are validated using TCGA cohort. For the DCSPs in AML, we assess the association between PAS and drug sensitivity and validate it in the BeatAML cohort. More details are described in the Materials and Methods section.

**Figure 2:**
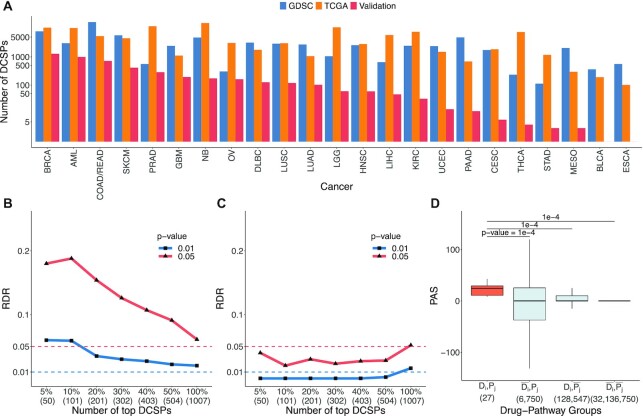
(A) The number of DCSPs identified in the GDSC cohort and TCGA cohort. For each cancer on the x-axis, the left-most (blue) barplot represents the results of the GDSC cohort, the middle (orange) barplot shows the number of DCSPs of the TCGA cohort, and the right-most (red) barplot is the number of validated DCSPs. The y-axis is presented in log_2_ scale and the cancers on the x-axis are ordered by their number of validated DCSPs. (B, C) The rediscovery rate (RDR) of DCSPs in terms of the association between PAS and drug sensitivity in AML. RDR is the proportion of the top 5%, 10%, 20%, 30%, 40%, 50%, and 100% of DCSPs identified in the discovery set (GDSC cohort) that is significant in the validation set (BeatAML cohort). (B) RDR of DCSPs with negative correlations and (C) RDR of DCSPs with positive correlations. The horizontal dashed lines present the target lines for the target levels of the *P* value at α = 0.05 (red) and 0.01 (blue). (D) PAS of Martens–PML–RARA (*P*_*j*_) druggable by quizartinib (*D*_*i*_) from the GDSC cohort in comparison with PASs of the following 3 groups: (i) same pathway but different drugs ($\overline{D_i}$, *P*_*j*_), (ii) same drug but different pathways (*D*_*i*_, $\overline{P_j}$), and (iii) different drugs and different pathways ($\overline{D_i}$, $\overline{P_j}$). *P*values of the permutation test are presented at the top of each pair. $\overline{D_i}$ represents the set of other drugs, while $\overline{P_j}$ refers to the set of other pathways. The values in parentheses of the x-axis are the numbers of samples for each group. The y-axis presents PAS values of the groups.

From all drug–pathway combinations across 23 cancers and 251 drugs in the GDSC cohort, we put together a total of 250,479 DCSP candidates. Among these, we identify 69,986 DCSPs with *t* statistics and a false discovery rate (FDR) <0.01 and those within the first quartile of χ^2^ statistics. Supplementary Figure S1A displays the distributions of the statistics of these DCSPs. Among these cancers, colon/rectum adenocarcinoma (COAD/READ) has the largest number of DCSPs (17,057; 24,37%), followed by breast cancer (BRCA), skin cutaneous melanoma (SKCM), and pancreatic adenocarcinoma (PAAD), with more than 4,000 (>5%) DCSPs (see Fig. 2A). In contrast, some cancers report only a few DCSPs, for example, 112 and 233 for stomach adenocarcinoma (STAD) and thyroid carcinoma (THCA), respectively. Details of the numbers and proportions of DCSPs identified in individual cancers are provided in Supplementary Table S2 and Supplementary Figure S2. All DCSPs are available on the study website [27].

### Validation of DCSPs in the TCGA cohort

Using the same computational procedure, among the 69,986 DCSPs identified in the GDSC cohort, 4,794 DCSPs are validated in the TCGA cohort. Fig. 2A shows the number of validated DCSPs for each cancer using the TCGA cohort; details are mentioned in Supplementary Table S2. BRCA has the largest number of validated CSPs (1,284), followed by AML (992). However, the validation rate of BRCA is relatively low (0.16) in comparison to AML (0.33), prostate adenocarcinoma (PRAD) (0.51), and ovarian cystadenocarcinoma (OV) (0.53). The numbers of validated DCSPs of PRAD (284) and OV (160) are about 5 times less than the one of BRCA. These diseases also have the top validation rates, while the other diseases have a small validation proportion of less than 20%. The number of validated DCSPs and the validation rate of individual cancers are provided in Supplementary Table S2. The number of DCSPs found in the TCGA cohort (*n* = 110,400) is higher than that in the GDSC cohort (*n* = 69,986). There are several possible reasons for the difference: the GDSC cohort contains data from cell lines, which tend to be more homogeneous compared to the patient-derived data from the TCGA cohort. Furthermore, the number of samples of each cancer in the TCGA cohort is much higher than that in the GDSC cohort (Supplementary Table S1), which increases the sensitivity in the test of differences.

### Correlation between PAS and drug sensitivity

Next we investigate the correlation between PAS and drug sensitivity in AML, the disease with a high validation rate and for which there exist extensive drug response assays in multiple datasets. Drug sensitivity is measured in terms of area under the curve (AUC) of cancer cell survival as a function of drug dose. A small AUC indicates a good drug response (i.e., the drug kills the cancer cells at the low end of the dose range). A negative correlation cor(PAS, AUC) means high PAS is associated with better drug response. This happens if the drug is effective in killing the cancer cells and the pathway *P* mediates the drug response. Such an observation would support our main hypothesis that cancers with activated druggable cancer-specific pathways are likely more responsive to the relevant drug. If there is no correlation, it is either because the drug is not effective (e.g., there is drug resistance) or because its effect is mediated by other pathways. A positive correlation means that higher PAS is associated with worse drug response, or lower PAS with better response, which is the opposite to our hypothesis. So our hypothesis would imply no positive correlation, and we consider this part as a negative control. Further details are in the Materials and Methods. Data from the BeatAML cohort are used for validation. Figs. 1D and E present an example of the involvement of quizartinib, pathway Martens–PML–RARA [20], and AML, where the correlation between PAS and AUC is −0.14 in the GDSC cohort and −0.20 in the BeatAML cohort. From all identified DCSPs for AML in the GDSC cohort, we collect 1,007 DCSPs that share 56 overlapped drugs with the BeatAML cohort. PASs of these DCSPs are also calculated in the BeatAML cohort. The DCSPs are first ranked by the correlation cor(PAS, AUC) in the GDSC. This rank is also used later for the results shown in Fig. 2. We assess the validation by computing the rediscovery rate (RDR), defined as the proportion of the top-ranking DCSPs identified in GDSC that have significant cor(PAS, AUC) in the validation set (BeatAML). DCSPs with *P* value <α are considered as significant, using target levels α = 0.05 and 0.01.

Fig. 2B presents the RDRs of the set of DCSPs with negative correlations. Here, the x-axis represents 5%, 10%, 20%, 30%, 40%, 50%, and 100% of top-ranking DCSPs in the discovery set (GDSC); the y-axis represents the corresponding RDRs at 0.05 (red line) and 0.01 (blue line) thresholds. Both RDR curves generally slope downward when the number of top DCSPs increases and closely reaches the target (horizontal dashed lines) at top 100% (the full set). From top 5% to top 20% of the red line, RDRs archive the highest value at ∼0.20. Supplementary Table S3 shows 28 DCSPs at top 20% that are rediscovered in the validation set. Our analyses of the cor(PAS_*u*_, AUC) and the cor(PAS_*d*_, AUC) of these 28 DCSPs show that the downstream pathway activation should be uninformative toward drug response (data not shown). Fig. 2C presents the RDRs for the set of DCSPs with positive correlations. The results show that most RDRs are close to the target lines (the horizontal dashed lines in the figure), supporting our expectation that there are no DCSPs where lower PAS is associated with better drug response.

Fig. 1B (with extension in Supplementary Fig. S3A) illustrates PASs of a top DCSP (ranked based on *t* statistics) of AML versus other cancers in the GDSC cohort. This AML-specific DCSP is the Martens_bound_by_PML_RARA_fusion (Martens–PML–RARA), which is druggable by quizartinib. Median PAS of AML (24.3) is 2.5 times greater than that of the remaining cancers (median = 9.7). The pattern is validated in the TCGA cohort (see Fig. 1C with extension in Supplementary Fig. S3B). The pathway was first described by Martens and colleagues [20] in the study on genes with promoters occupied by the PML–RARA fusion in APL, a well-studied subtype of AML disease [28]. Intriguingly, quizartinib is a small-molecule receptor tyrosine kinase inhibitor that targets FLT3 genes and has been shown to work for FLT3-mutated AML cases [29]. The FLT3 mutation is the one of the most common mutations in AML, which can be caused by the internal tandem duplication of FLT3 (FLT3–ITD), point mutations, and indels in the tyrosine kinase domain (FLT3–TKD) [30]. Among patients with APL, 47.9% carry FLT3 mutations [31], and it has been shown that PML–RARA fusion can collaborate with FLT3 mutation to induce an APL-like disease in the mouse [32].

We then investigate the correlation between the downstream activation and the drug sensitivity. Here PAS is defined as PAS_*d*_ – PAS_*u*_, so a high positive PAS corresponds to the downstream part of the pathway having higher activation relative to the upstream part. A similar procedure is applied for this version of PAS to compute the RDRs. Supplementary Figs. S4A and B show the RDRs of the set of DCSPs with negative and positive correlations, respectively. The RDRs generally follow the *P*value target lines (0.05 and 0.01) closely, indicating there is no evidence of correlation between downstream activation with drug response.

### Specificity of DCSPs in AML

We further investigate the specificity of the identified DCSPs of AML using the case in Fig. 2C as an example. Given *D* the set of drugs and *P* the set of pathways from the DCSPs identified in AML, we define $\overline{D_i} = \lbrace D_m | D_m \in D, m \ne i\rbrace$ as the set of the other drugs. Similarly, $\overline{P_j}$ is defined as the set of other pathways. Suppose a DCSP is specified by a combination of drug *D*_*i*_ and pathway *P*_*j*_ in AML. Then we investigate the overexpression of its PASs in comparison to PAS of these 3 other sets: (i) the same pathway but different drugs ($\overline{D_i}$, *P*_*j*_), (ii) the same drugs but different pathways (*D*_*i*_, $\overline{P_j}$), and (iii) different drugs and different pathways ($\overline{D_i}$, $\overline{P_j}$). To compare the PASs of group (*D*_*i*_, *P*_*j*_) with another group, we use a permutation test where the null distribution of the *t* statistic is generated by random permutation of cell-line labels. A total of 10,000 permutations are carried out to build the null distribution. Then, the actual *t* statistic and the population of the *t* statistics from the permuted dataset are used to calculate the empirical *P* values.

Fig. 2D presents the results of the permutation test for quizartinib and the Martens–PML–RARA combination [20] from the GDSC cohort. The results show that PASs of this DCSP (group [*D*_*i*_, *P*_*j*_]) are significantly higher than those of the groups of different pathways or both drugs and pathways ((*D*_*i*_, $\overline{P_j}$) and [$\overline{D_i}$, $\overline{P_j}$]; *P* = 1e-4), indicating that quizartinib is more closely linked to the Martens–PML–RARA pathway compared to the other pathways. Compared to the group of the same pathway but different drugs, this DCSP also has significantly higher PASs (*P* < 1e-4). Similar results are also observed for the other DCSPs of AML. The details are provided in Supplementary Table S4 and illustrated on the interactive website.

## Discussion and conclusion

To investigate the hypothesis that cancers with activated druggable cancer-specific pathways are more likely to respond to the relevant drugs, we have introduced PAS and applied it to conduct a systematic search of druggable cancer-specific pathways in 23 cancers from the GDSC cohort. The DCSPs of these cancers are then validated in the TCGA cohort. In support of the hypothesis, we observe a significant correlation between higher PAS and stronger drug response among the DCSPs identified in AML and validate this in the BeatAML cohort. All results are provided in an interactive website available to users.

PAS is defined to capture the druggability of a pathway for an individual cancer. In principle, this information can be used to build a model for predicting drug responses of tumors in precision medicine. Current models often apply black-box statistical and machine learning methods to multiple omics data to predict responses of a single drug (monotherapy) or combination of drugs (drug synergy) [33,34]. This sometimes makes the interpretation of the prediction models difficult [35]. One of the advantages of using PASs for the prediction model is its ability to keep track of the driving mechanisms through the pathway information. Furthermore, PASs can be applied to prediction in both monotherapy or drug synergy as long as the target gene list is collected from the drug(s).

This study has been conducted using the rich resources; however, the data still have some weaknesses. First, information on drug target genes is often incomplete, and off-target genes are generally unknown. We collect the target gene list provided from the GDSC cohort and extend with the curated information from the DrugBank database [22]. Recently, a community effort has been made to improve the target space of drugs via a web platform named Drug Target Commons [36]. Investigating the use of the drug target data of this database will be our future work. Second, the pathway databases are still incomplete, and we expect they will be improved in the future. Third, the number of cell lines of individual cancers in the GDSC is limited and could not be representative of the real data of the disease. Fourth, the GDSC and BeatAML cohorts share only a small number of drugs; this means a large number of DCSPs are not assessed in terms of drug response. This problem can be improved by producing more drug data. Despite the limited sharing drugs, integration of the 2 cohorts is considered in some recent studies. For example, Jafari and colleagues [19] propose bipartite network models to search for combination therapies in AML using the data from both the GDSC cohort and the BeatAML cohort . However, they did not focus on identifying potential biological pathways related to drug response. Finally, there is general lack of publicly available drug data of other cancers for validation.

## Materials and methods

### Functional network connectivity between driver genes, pathway, and target genes of drugs

To achieve the weights for PAS using the interaction between driver genes, pathway, and drug target genes, we utilize the network enrichment analysis (NEA) [21]. Briefly, NEA originally assesses the functional network connectivity between 2 gene sets: a functional gene set (FGS; e.g., driver alteration) and an altered gene set (AGS) associated with a certain downstream biological state (e.g., differentially expressed [DE] genes). Compared to the traditionally used gene-set enrichment analyses (GSEAs) [37], NEA extends GSEA with topological information in terms of gene interaction networks, which provide a biologically informative category. A comprehensive network containing 1,445,027 functional links between 16,299 distinct HUPO genes is considered in the analysis.

In the current application, NEA is applied for 3 pairs of gene sets, including driver genes, pathway genes, and drug target genes. For each pair, 1 gene set is selected for FGS and the remaining gene set is for the AGS. In particular, FGS is assigned for the set of drug target genes in (drug target genes, pathway genes) and (drug target genes, driver genes) while for (driver genes, pathway), the driver genes are used for FGS. Finally, NEA simplifies the assessment of the functional connectivity by defining an enrichment score as

(1) \begin{eqnarray*}
z = \frac{d_{AF} - \overline{d}_{AF}}{\sigma _{AF}}
\end{eqnarray*}

where *d*_*AF*_ is the number of connected links between AGS and FGS, and $\overline{d}_{AF}$ and σ_*AF*_ are the mean and standard deviation of *d*_*AF*_, respectively, which are estimated on a randomized network under the null hypothesis. Thus, for each PAS, we collect 3 corresponding *z* scores expressing the overrepresentation of drug target genes on cancer driver genes (*z*_1_), driver genes on pathway genes (*z*_2_), and target genes on pathway genes (*z*_3_) based on the functional gene network. Finally, these 3 enrichment scores are then converted into normal probability scores (*w*_1_, *w*_2_, and *w*_3_), which are used as the weights for PAS.

### Discoveries of druggable cancer-specific pathways

Given a drug *D*_*i*_, a pathway *P*_*j*_ is considered as specific to a cancer *C*_*z*_, that is, DCSP, if the pathway overactivates in that cancer while activation scores of this pathway in other cancers are not significantly different from each other (see Fig. 1B). The issue is straightforward: if we consider only 2 cancers, a standard statistical approach such as a *t*-test can be applied directly to PASs. However, when there are more than 2 cancers (e.g., 23 different cancers from the GDSC cohort in this study), the standard method only ensures that a cancer is different from the rest, but the remaining cancers might be different from each other. Therefore, in this case, the specificity of the pathway for the remaining cancers is not guaranteed. To identify the DCSPs, we apply a 2-statistic approach originally developed in a recent study [38] for the PAS data of the GDSC cohort. The method provides 2 statistics for each cancer: (i) a robust *t*-test (*T*_1_) for comparing between that cancer and the rest and (ii) a χ^2^ statistic (*T*_2_) for jointly comparing the remaining cancers.

For an activated pathway that is cancer specific, we expect a large *t*statistic for *T*_1_ and a small χ^2^ statistic for *T*_2_. To account for multiple testing, the FDRs [39] of *T*_1_ are calculated, and we keep DCSPs with FDR <0.01. We further keep only DCSPs whose χ^2^ statistics are within the first quartile. Finally, we apply the following sample size conditions: (i) for each DCSP, the number of samples for each supporting cancer is larger than 5, and (ii) it is supported by at least 3 cancers.

### Pathway activation score in relation to drug response

Our hypothesis is supported if the pathway *P*_*j*_ mediates the response to drug *D*_*i*_ in cancer *C*_*z*_; statistically, this is the case if the pathway activity of DCSP(*D*_*i*_, *P*_*j*_, *C*_*z*_) correlates with the drug response. Fig. 1D shows an example in AML of the relation between PAS and the AUC of drug sensitivity of the pathway Martens–PML–RARA [20] druggable by quizartinib, where the AUCs are obtained from cell lines actually treated with quizartinib. Given a DCSP(*D*_*i*_, *P*_*j*_, *C*_*z*_), we first apply the normal score transformation on both PAS and drug sensitivity (AUC) of the tumors in cancer *C*_*z*_. Subsequently, we calculate the Pearson correlation between PAS and AUC as cor(PAS, AUC). Here, 2 versions of PAS for upstream and downstream activation are used to compute cor(PAS, AUC). The PAS of the upstream version is defined as PAS = PAS_*u*_ – PAS_*d*_, while for the downstream version, PAS = PAS_*d*_ – PAS_*u*_. As activation, only positive values are considered.

### Datasets

We use the GDSC cohort as the discovery set. Validation sets have been obtained from the following sources: (i) TCGA cohort, (ii) Therapeutically Applicable Research to Generate Effective Treatments (TARGET) cohort, and (iii) BeatAML cohort.

#### GDSC dataset

The GDSC project [14] has been undertaken with the aim of discovering cancer biomarkers that are highly responsive to anticancer drugs. This cohort contains the genomic information of more than 1,000 human cancer cell lines and drug sensitivities of more than 250 drugs.

The drug data from the GDSC cohort (version 17.3) contain a total of 224,202 cell line–drug experiments from 251 drugs and 1,065 cell lines. We use only 125,894 monotherapy profiles of 684 cell lines from 23 cancers after removing the profiles with more than 1 replicate. The number of cell lines of a cancer ranges from 6 to 64; AML has 28 cell lines. The potential driver genes of the samples, including mutations and fusion genes, are collected from Depmap Portal [40]. We keep mutations with an occurrence at least 2% of total samples across cancers. For the fusion genes, we keep all fusions with at least 2 occurrences and overlapping with the fusions found in the Mitelman database [41]. The expression data of 17,715 genes from these cell lines are also achieved.

#### TCGA and TARGET datasets

TCGA [15] is led by the National Cancer Institute’s Center for Cancer Genomics and the National Human Genome Research Institute with the aim of providing a landscape of genomic characterization for more than 33 malignant diseases. TARGET is an ongoing project that provides the comprehensive genomic landscape targeted toward countering childhood cancer. In the validation step, we collect data of 22 cancers from the TCGA cohort and neuroblastoma (NB) from the TARGET cohort [42]. These cancers are matched with the cancers in the GDSC cohort of the discovery set. The data contain expressions of 37,636 genes from a total of 8,825 samples across 23 cancers. The detailed information of these cancers is provided in Supplementary Table S1. Gene expressions normalized by fragments per kilobase of transcript per million mapped reads originally reported from the cohorts are converted to transcript per million (TPM) for downstream analyses. Mutations and fusion genes are also collected and filtered to obtain potential driver genes with high occurrence. Frequent mutations with occurrence of at least 1% of total samples are kept, and the same filter in the GDSC cohort is applied for fusion genes.

#### BeatAML dataset

BeatAML [16] is an ongoing project that aims to provide an extensive landscape of AML, comprising clinical, genomic, and drug response data. This cohort contains RNA sequencing samples of 461 AML cases. These samples are sequenced by the Illumina HiSeq 2500 platform (100-bp paired-end reads) after processing with the Agilent SureSelect Strand-Specific RNA Library Preparation Kit on the Bravo robot. The FASTQ files of these samples are input to XAEM [43]; then, expressions in TPM of 26,086 genes are collected. After removing unexpressed genes (TPM ≤ 1*e* − 2 in more than $90\%$ of samples), 23,035 genes remain. The mutations and fusion genes collected from the BeatAML cohort are used. The fusion genes are filtered by the same procedure in the GDSC cohort. The drug sensitivities of 122 compounds reported in terms of both IC_50_ and AUC are also collected. The data consist of 47,650 records from 528 patients with AML.

The results of this study are available on the DCSP website [27].

## Data Availability

The implementations of the PAS generation and the shiny application are available on the DCSP website [27]. All related datasets can be downloaded from a public Zenodo repository [44]. All supporting data and materials are available in the *GigaScience* respository, GigaDB [45].

### Additional Files

**Supplementary Table S1**. List of 23 cancers of the GDSC cohort using in this study. Note that COAD and READ in the TCGA cohort are combined together to be consistent with the GDSC cohort.

**Supplementary Table S2**. The DCSPs discovered from the GDSC cohort and the TCGA cohort across 23 cancers. The validated DCSPs are the DCSPs identified in the GDSC cohort and rediscovered in the TCGA cohort. The last column presents the proportion of validated DCSPs (PV) in the TCGA cohort.

**Supplementary Table S3**. List of 28 DCSPs responding to anticancer drugs in the GDSC cohort and the BeatAML cohort.

**Supplementary Table S4**. PAS of pathway *P*_*j*_ treated by drug *D*_*i*_ in comparison with PASs of following 3 groups: (i) same pathway but different drugs ($\overline{D}_i,\ P_j$), (ii) same drug but different pathways ($D_i, \overline{P}_j$), and (iii) different drugs and different pathways ($\overline{D}_i, P_j$). The values in the last 4 columns are the median PAS of the groups. *P*values of the permutation tests are reported in parentheses.

**Supplementary Fig. S1**. The *t* statistics and χ^2^ statistics of DCSP candidates from the GDSC cohort: (A) across 23 cancers and (B) AML.

**Supplementary Fig. S2**. The proportion of identified DCSPs for individual diseases.

**Supplementary Fig. S3**. PASs of Martens–PML–RARA druggable by quizartinib in the (A) GDSC cohort and (B) TCGA cohort. The orange box plots represent PAS of AML, and the green ones represent PASs of other cancers.

**Supplementary Fig. S4**. The rediscovery rate (RDR) of DCSPs in terms of the association between PAS and drug sensitivity in AML. RDR is the proportion of the top DCSPs identified in the GDSC cohort that is significant in the BeatAML cohort. Here, PAS is defined as *PAS*_*d*_ − *PAS*_*u*_, so a higher value of PAS indicates a higher activation of the downstream part than the upstream part. (A) RDR of DCSPs with negative correlations and (C) RDR of DCSPs with positive correlations. The horizontal dashed lines present the *P* value target lines (0.05 and 0.01). The RDRs follow the null target lines (0.05 and 0.01), that is, null results, indicating that there is no evidence of drug response when the pathway has higher activation downstream rather upstream of the drug targets.

## Abbreviations

AGS: altered gene set; AML: acute myeloid leukemia; APL: acute promyelocytic leukemia; AUC: area under the curve; DCSP: druggable cancer-specific pathway; FDR: false discovery rate; FGS: functional gene set; GDSC: Genomics of Drug Sensitivity in Cancer; GSEA: gene-set enrichment analysis; NEA: network enrichment analysis; PAS: pathway activation score; PAS_*u*/*d*_: pathway activation score for up/downstream activity; RDR: rediscovery rate; TARGET: Therapeutically Applicable Research to Generate Effective Treatments; TCGA: The Cancer Genome Atlas; TPM: transcript per million.

### Competing Interests

The authors declare no competing interests.

### Funding

This work was partially supported by funding from the KI Research Foundation, the Swedish Research Council (VR), and the Swedish Foundation for Strategic Research (SSF).

### Authors’ Contributions

TNV and YP initiated and oversaw the study. QTT, TNV, and YP contributed to method development and manuscript writing. QTT, TZ, and TNV performed the bioinformatics analysis and webpage development with input from YP.

## Supplementary Material

giac091_GIGA-D-22-00079_Original_Submission

giac091_GIGA-D-22-00079_Revision_1

giac091_GIGA-D-22-00079_Revision_2

giac091_GIGA-D-22-00079_Revision_3

giac091_Response_to_Reviewer_Comments_Original_Submission

giac091_Response_to_Reviewer_Comments_Revision_1

giac091_Response_to_Reviewer_Comments_Revision_2

giac091_Reviewer_1_Report_Original_Submission Jing Tang -- 5/17/2022 Reviewed

giac091_Reviewer_1_Report_Revision_1 Jing Tang -- 8/2/2022 Reviewed

giac091_Reviewer_2_Report_Original_Submission Sebastian Vosberg -- 5/19/2022 Reviewed

giac091_Reviewer_2_Report_Revision_1 Sebastian Vosberg -- 7/29/2022 Reviewed

giac091_Supplemental_File
