# Supplementary material for: Discovery of druggable cancer-specific pathways with application in acute myeloid leukemia
Source: Gigascience. 2022 Sep 29;11:giac091. doi: 10.1093/gigascience/giac091 (PMC9520771; doi:10.1093/gigascience/giac091)
Supplement: giac091_GIGA-D-22-00079_Revision_1 [file giac091_giga-d-22-00079_revision_1.pdf]

GigaScience

Discovery of Druggable Cancer-Specific Pathways with Application in Acute Myeloid Leukemia

--Manuscript Draft--

**Manuscript Number:**	GIGA-D-22-00079R1	
**Full Title:**	Discovery of Druggable Cancer-Specific Pathways with Application in Acute Myeloid Leukemia	
**Article Type:**	Research	
**Funding Information:**	KI Research Foundation	Not applicable
	Swedish Research Council (VR)	Not applicable
	Swedish Foundation for Strategic Research (SSF)	Not applicable
**Abstract:**	An individualized cancer therapy is ideally chosen to target the cancer's driving biological pathways, but identifying such pathways is challenging because of their underlying heterogeneity and there is no guarantee that they are druggable. We hypothesize that a cancer with an activated druggable cancer-specific pathway (CSP) is more likely to respond to the relevant drug.<br><br>In this study we develop and validate a systematic method to search for such CSPs, by (i) introducing a pathway activation score (PAS) that integrates cancer-specific driver mutations and gene expression profile, and drug-specific gene targets; (ii) applying the method to identify CSPs from pan-cancer datasets; (iii) analysing the correlation between PAS and the response to relevant drugs. In total, 4,794 CSPs from 23 different cancers are discovered in the Genomics of Drug Sensitivity in Cancer database and validated in The Cancer Genome Atlas database. Supporting the hypothesis, for the CSPs in acute myeloid leukemia, cancers with higher PASs are shown to have stronger drug response, and this is validated in the BeatAML cohort. All CSPs are publicly available at https://www.meb.ki.se/shiny/truvu/CSP/.	
**Corresponding Author:**	Trung Nghia Vu<br>Karolinska Institutet<br>Stockholm, Stockholm SWEDEN	
**Corresponding Author Secondary Information:**		
**Corresponding Author's Institution:**	Karolinska Institutet	
**Corresponding Author's Secondary Institution:**		
**First Author:**	Quang Thinh Trac	
**First Author Secondary Information:**		
**Order of Authors:**	Quang Thinh Trac	
	Tingyou Zhou	
	Yudi Pawitan	
	Trung Nghia Vu	
**Order of Authors Secondary Information:**		
**Response to Reviewers:**	We have prepared point-by-point responses to specific reviewer and editor comments, however the responses contain some figures which are not able to included in this "Respond to Reviewers" box of the submission system. Therefore, we concatenated the response letter to the main text in a single file. Please see the details of the responses in the Manuscript file.	
**Additional Information:**		

Powered by Editorial Manager® and ProduXion Manager® from Aries Systems Corporation

Question	Response
Are you submitting this manuscript to a special series or article collection?	No
**Experimental design and statistics**<br><br>Full details of the experimental design and statistical methods used should be given in the Methods section, as detailed in our Minimum Standards Reporting Checklist. Information essential to interpreting the data presented should be made available in the figure legends.<br><br>Have you included all the information requested in your manuscript?	Yes
**Resources**<br><br>A description of all resources used, including antibodies, cell lines, animals and software tools, with enough information to allow them to be uniquely identified, should be included in the Methods section. Authors are strongly encouraged to cite Research Resource Identifiers (RRIDs) for antibodies, model organisms and tools, where possible.<br><br>Have you included the information requested as detailed in our Minimum Standards Reporting Checklist?	Yes
**Availability of data and materials**<br><br>All datasets and code on which the conclusions of the paper rely must be either included in your submission or deposited in publicly available repositories (where available and ethically appropriate), referencing such data using a unique identifier in the references and in the "Availability of Data and Materials" section of your manuscript.	Yes

Powered by Editorial Manager® and ProduXion Manager® from Aries Systems Corporation

Have you have met the above requirement as detailed in our Minimum Standards Reporting Checklist?	

Powered by Editorial Manager® and ProduXion Manager® from Aries Systems Corporation

Responses and maintext
Click here to access/download;Manuscript;csp_gigascience_response_mainte

Response to reviewers

Editor's comments:

Your manuscript "Discovery of Druggable Cancer-Specific Pathways with Application in Acute Myeloid Leukemia" (GIGA-D-22-00079) has been assessed by our reviewers. Although it is of interest, we are unable to consider it for publication in its current form. The reviewers have raised a number of points which we believe would improve the manuscript and may allow a revised version to be published in GigaScience.

Response to editor: *Thank you for giving us opportunity to improve our study and submit the revision of the manuscript. We have carefully addressed all concerns of the reviewers as explained in the point-by-point response below.*

Reviewer 1 comments:

Discovery of druggable cancer-specific pathways with application in AML. Authors developed a method to identify druggable cancer-specific pathways (CSPs). Pathway activity score (PAS) is measured by gene expressions and then weighted by the functional connectivity as well as relatedness to cancer driver genes and drug targets. Drugs that can target these pathways are found to be more sensitive, inferred from the GDSC cancer cell line data and then validated in the BeatAML cohort. The analysis involves an integration of multiple data types from multiple resources across cell lines to patient-derived samples. I think the topic fits the scope of the journal and the conclusion is generally valid. However, some major comments are given to improve the clarity.
Response to reviewer: *We thank the reviewer for his/her many constructive comments that have improved our manuscript.*

Major comments:

1. Rationale of separating upstream versus downstream genes. Why "less or no response" of a drug is expected "if the driver gene is downstream of the target"? I would assume that the opposite is true.

Response to reviewer: *This is an important point that has led us to perform more analysis. Our hypothesis was that for a drug to be effective on a tumor, part of the pathway containing the target genes is highly activated, where high activation is measured relative to the other part of the pathway. This was the reason we considered positive PAS_u-PAS_d as the score for pathway activation. But in view of the reviewer's comment, we realized that we also need to show evidence that the downstream activation is not informative of drug response. As a motivation, suppose the driver is downstream of the target and that part of the pathway (PAS_d) is highly activated, while the target gene and its upstream genes (PAS_u) are not activated. Then, intuitively, in this case an*

inhibitor will not have a chance to work. This is the case where PAS = PAS_d - PAS_u > 0. We have now investigated this version of PAS and followed the same analytical steps as for the original PAS. Indeed, we found that it is not correlated with drug response; see Figure 1R below. We have added this result in the manuscript (the last paragraph of section Correlation between PAS and drug sensitivity) and revised the text in the second paragraph of section Pathway Activation Score.

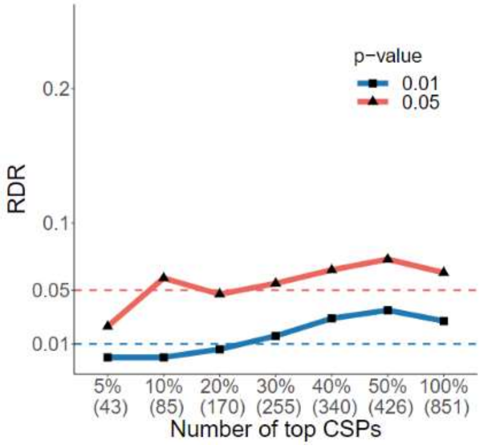

Figure 1R: The rediscovery rate (RDR) of the CSPs in the validation set, among the top CSPs with negative correlation between PAS and AUC in the training set. Here PAS is defined as PAS_d - PAS_u, so a high positive PAS corresponds to the downstream part of the pathway having higher activation relative to the upstream part. The RDRs follow the P-value target lines (0.05 and 0.01) closely, indicating null results. This means that there is no evidence of drug response when the pathway part downstream of the drug target has higher activation than the upstream part.

2. The PAS is calculated as the product of the gene expression values and their weights. It seems to me that the PAS will favor highly expressed genes, which may not be directly correlated to cancer-specific or drug-specific pathways.

Response to reviewer: *The value of PAS is first computed as the sum of the expression of the genes in the pathway, so it depends on the whole pathway rather than just a few highly expressed genes. For a cancer-specific pathway, by construction, the PAS of the cancer indeed has a higher score than that of other cancers, since we are searching only among activated pathways.*

The drug specificity is taken into account via the weights of the PAS calculated from the connectivity between drug-target genes, driver genes, and pathway genes. Furthermore, the gene sets used to calculate the PAS are not random but specific to individual drugs through the collection of the upstream and downstream genes relative to the target genes of the drug.

3. Difference between cancer-specific pathways or drug-specific pathways. It seems that the pathways are for a specific cancer-drug pair (e.g. Figure 1A), but how to predict drugs from these pathways?

Response to reviewer: *Actually, the drugs are not predicted from the pathways, but we have defined PAS based on the information of both a pathway and a specific drug. So, by construction, a CSP has both pathway- and drug-specificity, but it seems unwieldy to put both in the name.*

4. Figure 1A and C are not consistent, as the average of PAS for AML is around 4.5 in Figure1A, but in Figure1C the average is around 0.

Response to reviewer: *Figures 1B and 1D (previously Figures 1A and 1C) have the same PAS data but on different scales. In Figure 1D, we applied the normal-score transformation to the original PAS values in Figure 1B. We use the transformation for both PAS and AUC (in Figure 1D) so that we can use the standard Pearson correlation. We described the transformation in the section Pathway activation score in relation to drug response - Materials and Methods.*

5. The 251,615 candidates are meant for candidate pathways?

Response to reviewer: *We are sorry for the confusion. The number refers to the total possible CSP candidates that we obtain from all cancers in the GDSC dataset. We have revised the text to improve clarification in section Identification of cancer-specific pathways.*

6. Among the cancer types, colon cancer has the largest number of CSPs, does it imply that the drug sensitivity on colon cancers will be higher than the other cancer types? or it means that gene expressions of these CSPs are higher than in colon cancer than the other cancer types?

Response to reviewer:

We do not observe a clear relationship between the largest number of CSPs in colon cancer and the gene expression or drug sensitivity.

To investigate if the drug sensitivities of colon cancer are higher than other cancer types, we collect the drug data of the colorectal cancers (COAD/READ), breast cancer (BRCA) and acute myeloid leukemia (AML) (top cancers with the number of validated CSPs), and the remaining cancers; see Figure R2. The figure shows that there is no significant difference between the colon cancer and the breast cancer, but the colon cancer has slightly higher AUC (or lower drug response) compared to AML and the others.

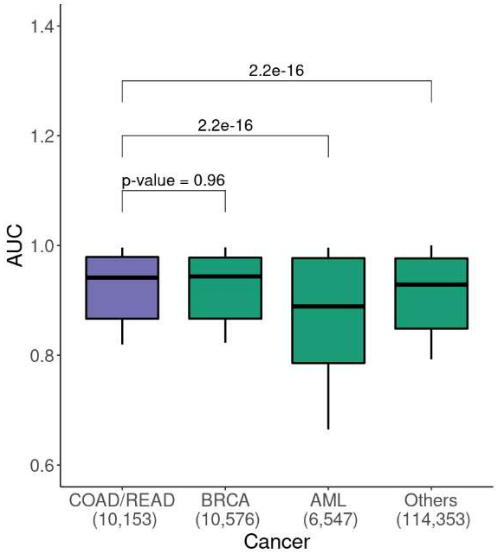

Figure R2. Drug sensitivity of COAD/READ, BRCA, AML, and other cancers in terms of AUC

To investigate the difference of the gene expression of the CSPs in colorectal vs other cancers, for each CSP of COAD/READ, we collect the gene expression of three gene sets of its pathway: up-stream genes (Up), down-stream genes (Down), and all (Up + Down). We then calculate the fold-change of the sum of gene expression of the three gene sets between COAD/READ vs the others. Figure R3 presents the distributions of the fold-change of gene expression between COAD/READ and other cancers in the three gene sets. The figure shows that the fold change of expression in all three plots are around one (median=1), indicating that there is no difference between the gene expression of colorectal vs other cancers.

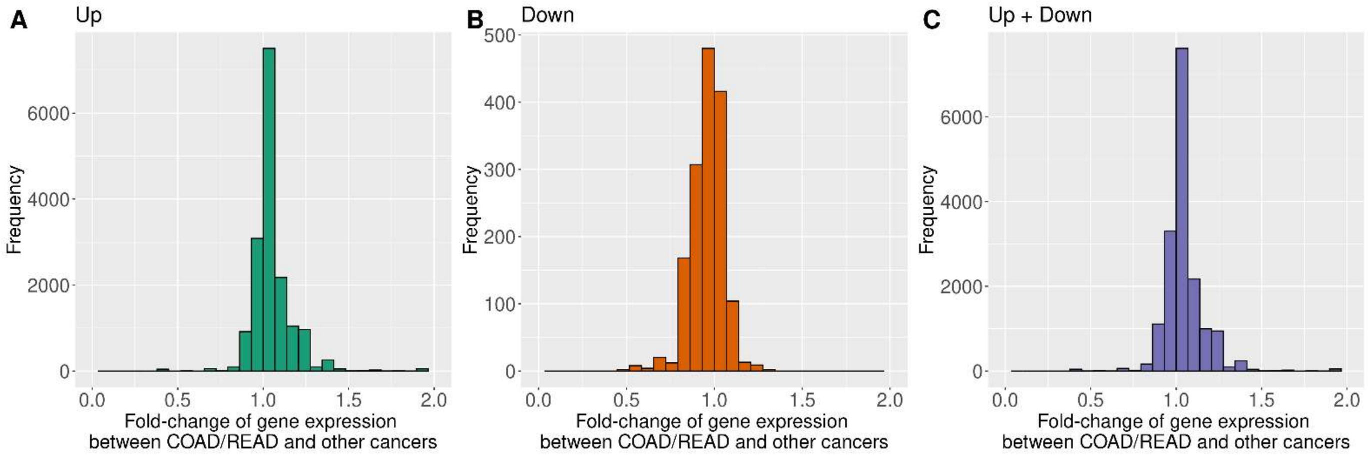

Figure R3. Comparison of gene expression between colon cancers (COAD/READ) vs the other cancers. Panels A, B, and C present the results for the gene sets of Up, Down, and all (Up + Down), respectively. The fold-change between two groups is calculated based on the sum of gene expression of the genes in the gene set

7. Figure 2D. the y-axis is not log2 scale as described in the figure caption.

Response to reviewer: *Thank you for pointing out this. The caption of Figure 2D have been corrected.*

8. What does Figure S1 tell by correlating the two statistics?

Response to reviewer: *Figure S1 only presents the joint distributions of the two statistics, not to highlight the correlation between them. For an activated pathway to cancer specific, we expect to have a large t-statistic but a small chi2-statistic, which is discussed in section Discoveries of cancer-specific pathway - Materials and Methods.*

9. In Table S4, what are the other drugs that target the same pathways? Most of these drugs do not have a significant PAS compared to the putative drug. What might be the explanation for it?

Response to reviewer: *In Table S4, the cases of other drugs that target the same pathway belong to* $(\overline{D}_i, P_j)$ *group. If two drugs have a similar set of genes strongly linked to the pathway, they might have a comparable impact on the pathway. However, since drugs mostly have different sets of target genes, there is a large variability in the PASs of these drugs on the same pathway. This might explain the small number of drugs with significant PAS compared to the putative drug* D_i.

10. Recent studies on GDSC integration with beatAML could be reviewed in the introduction or discussion, e.g https://pubmed.ncbi.nlm.nih.gov/35440130/

Response to reviewer: *We have revised the section of Introduction and the section of Discussion and Conclusion with the suggested recent studies.*

Reviewer 2 comments:

Trac et al. provide a computational approach to identify cellular pathways that can be druggable using targeted treatment approaches. To this end, the authors compute "pathway activity scores" (PASs) for pathway/drug combinations, based on mRNA expression of pathway members across publicly available data sets and a priori information about the drug target to evaluate upstream and downstream gene expression individually. This approach is further extended by incorporating cancer entities to identify "cancer specific pathways" (CSPs). Finally, the results are set in context with data from high throughput drug sensitivity screens with a focus on data from acute myeloid leukemia (AML) samples.

Evaluating the gene expression of upstream and downstream pathway members with respect to druggable target genes is an interesting approach and can be informative in terms of treatment-associated pathway activity. Both the results and the approach itself are able to contribute to the field. Still, several issues need to be addressed in order to improve the quality of the manuscript:

Response to reviewer: *Thank you for your positive and constructive comments. We appreciate the thoughtful comments and have addressed them in our revision.*

Major issues:

1. Improve overall structure
  - approach and workflow are unclear in the beginning of the manuscript
  - describe individual steps in the beginning (Introduction), introduce specific terms accordingly
  - precisely describe the concepts of "PAS" and "CSP" when introducing them
  - clearly describe how "CSPs" are ranked when showing the results of RDR
  - please describe more clearly (also in the beginning) why in particular AML associated pathways are considered

Response to reviewer:

We have substantially revised the last paragraph of introduction section to clarify the approach, workflow, the terms of PAS and CSP and reasons for consideration of AML associated pathways in the beginning of the manuscript.

Results of RDR in Figure 2 are based on CSPs ranked by the correlation between PAS and AUC in the GDSC cohort. To clarify this, we have revised the second paragraph of section Correlation between PAS and drug sensitivity – Results.

2. Improve readability/understanding of the results
  - precisely describe what "PAS" refers to, i.e. PASs are computed per pathways/drug pair (as it is written in the Methods section)
  - precisely describe what "CSP" refers to, i.e. CSPs are computed per pathways/drug/cancer triplet (as it is written in the Methods section)
  - if CSP does not refer to a (druggable) pathway being specific to a particular (single!) cancer

entity, this should be renamed to avoid confusions
- in the text, several numbers are given without proper explanation where they are coming from (e.g. "251,615 candidates", "1051 CSPs")

Response to reviewer:

Thank you for pointing out these issues. We have improved readability by revising the Result section following the suggestions. The precise descriptions are included in early part of the results of PAS (section Pathway activation score) and CSP (section Identification of cancer-specific pathways). We use "CSP candidates" to separate from the identified druggable CSPs which pass the statistic conditions. Extra explanations for the numbers are also included.

3. Improve reproducibility:
a) unclear how "target genes" are defined, provide a list of target genes per drug (i.e. in table S3)
b) unclear how up-/downstream genes within gene sets (e.g. as defined within MSigDB) are defined, please describe
c) unclear how results were filtered based on correlation between PAS/AUC, please provide cutoffs
d) unclear what is meant by "For our definition of PAS, we do not expect positive correlation, so this part can be used as a negative control". How can that be used as a negative control? Please elaborate.
e) unclear what is meant with "target lines", to which the authors refer in Fig 2B/C

Response to reviewer:

a) We collected "target genes" from the GDSC cohort and extended with the curated information from the DrugBank database which was mentioned in the Discussion and Conclusion section. To improve readability, we have revised the last paragraph of the "Pathway activation score" section with the information. Furthermore, we uploaded the full list of target genes used to folder "geneset" at the GitHub site of this study: https://github.com/tracquangthinh/CSP

b) The upstream and downstream genes are defined as description in Figure 1A using multiple directed network databases as described in the section Pathway activation score. We have also uploaded the scripts and data to generate the up-/downstream genes to folder "geneset" at the GitHub site which can be reproducible.

c) The results of AML are not filtered based on a fixed threshold of the correlation between PAS and AUC. Instead, a threshold for p-value of the correlation (p-value $< \alpha$) is used to identify significant CSPs. In practice, we investigate the results for $\alpha=0.01$ and $\alpha=0.05$. The results are assessed further by the rediscovery rate (RDR) which presents the rate of top CSPs (ranked by the correlation) in the GDSC cohort which are also significant in the BeatAML cohort (Figures 2B-2C). We have highlighted this in the second paragraph of section Correlation between PAS and drug sensitivity – Results. To enhance the reproducibility, we have uploaded the source code for replicating the RDR computation to folder "rdr_calculation" at the GitHub site.

d) A positive correlation means that higher PAS is associated with worse drug response, or lower PAS with better response, which is opposite to our hypothesis. As we do not expect positive correlation, this part can be used as a negative control. We have clarified this in the first paragraph of section Correlation between PAS and drug sensitivity – Results.

e) Target lines in Figures 2B & 2C are the horizontal dashed lines representing the expected RDR if the results are null, drawn at two p-value thresholds (alpha = 0.05 and 0.01). In this study, we expect the RDR is above the dashed lines for the case of negative correlation between PAS and AUC (Figure 2B) and surrounding the dashed lines for the case of positive correlation (negative control, Figure 2C). We have revised the main text and the caption of the figure to improve the clarification.

4. Correct for inconsistencies/errors:
- labeling of Fig 1 is inconsistent. In the legend, no figure panels are described, while in the figure itself, some parts (still not all) are labelled with A/B/C/D. The left part (workflow) has no label, the "toy examples" do have. Please use consistent labeling throughout the figure, legend, and text
- labeling of Fig 2 is incomplete/misleading
  - please provide information which data is shown in Fig 2D (GDSC?)
  - please rephrase "CSPs of the same pathway", doesnt make sense. Will be obsolete if "CSP" is renamed (see comment above)
- Fig 2: displaying the numbers of CSPs per entity on a log scale is not appropriate and misleading! this should be given in absolute numbers. The authors may consider to describe the validation as relative numbers in an independent figure panel to improve readability.

Response to reviewer:

We have revised Figure 1 to improve the consistencies. Particularly, the left part including the workflow and the 'toy example' is separated as a panel and assigned now with label A, the names of other panels are changed accordingly. Two labels "Gu" and "Gd" indicating to the up and down-stream gene sets of the toy example, which are not at the same level of the panels of the figure. Now, we have changed the labels ("Gu" and "Gd") to italic style and moved them to the bottom-right corners to avoid the confusion. Furthermore, the caption of Figure 1 has also revised to match the description of each panel.

Indeed the data of Figure 2D are from GDSC; we have updated the caption of Figure 2D for clarification. We have also excluded "CSP" from the name of the groups (such as "CSPs of the same pathway"). Regarding the scale of Figure 2A, we feel that for count data with a big dynamic range (orders of magnitude difference) log scale is better for presentation; the absolute numbers tend to exaggerate differences between large numbers while hiding differences between small numbers. We also see that separating the validation data into an independent panel does not improve clarity.

Minor issues:

(5) please elaborate on the description/discussion of results:

- can the authors elaborate on the differences in total CSPs found in the GDSC and TCGA data sets, as there are substantial differences?

Response to reviewer:

The total of CSPs found in the TCGA cohort (n = 110,400) is indeed higher than that in the GDSC cohort (n = 69,986). There are several possible reasons for the difference: The GDSC cohort contains data from cell lines, which tend to be more homogeneous compared to the patient-derived data from the TCGA cohort. Furthermore, the sample sizes in the TCGA cohort are much higher than those in the GDSC cohort (Supplementary Table S1), thus increasing the sensitivity in the test of differences. We have updated the section Validation of CSPs in the TCGA cohort - Results.

- have the authors elaborated on different mutations within target genes? The authors describe the FLT3 as "The FLT3 mutation is the one of the most common mutations in AML caused by the internal tandem duplication of FLT3", which is only partly true. There are additional mutations in the gene which occur frequently in AML, with different impact on the protein. This should be at least considered in the text.

Response to reviewer:

In PAS, different mutations within the target genes are allowed. Those mutations are taken into account in the connectivity weight w_1 between driver genes and target genes. For the FLT3 gene, indeed, besides FLT3-ITD there are other mutations of FLT3 such as point mutations and indels also occurring frequently in AML with different impact on the protein (PMID: 31821677). We have revised the relevant text in the last paragraph of section Correlation between PAS and drug sensitivity – Results.

- Please elaborate on the result that the PAS of the (Di,Pj) group and the (not-Di,Pj) group is significantly not different (Fig 2D) in more detail. The authors state that "This can be due to Martens-PML-RARA pathway in AML patients is also linked to other anti-AML drugs, not only quizartinib", but this pathway is not listed in combination with another drug in table S3.

Response to reviewer:

During the revision work, we have improved the pipeline for further clarity and reproducibility. This has led to some slight changes in the results; the manuscript has been updated with the new results. The previous Figure 2D was plotted in logscale, but the label shows original scale which was pointed out by Reviewer 1 above. We have fixed the issue in the new Figure 2D (see Figure R4 below, and in the new analysis PAS of the (Di,Pj) group is significantly different to all three remaining groups. We have updated the figure 2D and the related text in the second paragraph of section Specificity of CSPs in AML - Results.

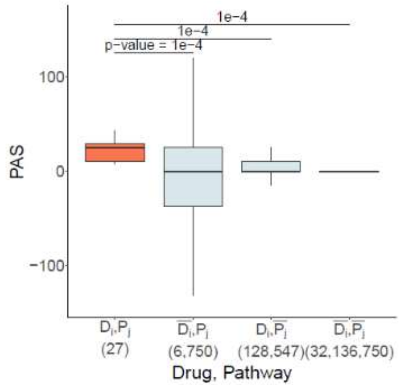

Figure R4: PAS of Martens-PML-RARA (Pj) druggable by quizartinib (Di) from the GDSC cohort in comparison with PASs of following three groups: 1) the same pathway but different drugs (not-Di, Pj), 2) the same drug but different pathways (Di, not-Pj), and 3) different drugs and different pathways (not-Di, not-Pj). P-values from the permutation test are presented on the top of each pair. Not-Di represents the set of other drugs, while not-Pj refers to the set of other pathways. The values in the parentheses of x-axis are the numbers of samples for each group. The y-axis presents PAS values of the groups.

(6) the last part of the last abstract about the "Martens-PML-RARA" pathway, quizartinib and FLT3 should be moved to the Results section.

Response to reviewer:

We have added the text about "Martens-PML-RARA" pathway, quizartinib and FLT3 in subsection "Correlation between PAS and drug sensitivity" in the Results section.

(7) please re-check the language, as there are several minor mistakes in wording and/or grammar. The authors may consider to have the manuscript read by a native speaker

Response to reviewer:

We have carefully revised the manuscript to fix the typos and improve the text. In addition, the manuscript has been proofread by a native English speaker.

Placeholder for OUP logo oup.pdf

Placeholder for journal logo gigascience-logo.pdf

GigaScience, 2022, 1–7

doi: xx.xxxx/xxxx
Manuscript in Preparation
Paper

PAPER

Discovery of Druggable Cancer-Specific Pathways with Application in Acute Myeloid Leukemia

Quang Thinh Trac[1], Tingyou Zhou[2], Yudi Pawitan[1] and Trung Nghia Vu[1,*]

[1]Department of Medical Epidemiology and Biostatistics, Karolinska Institutet, Nobels väg 12A, Stockholm 17177, Sweden and [2]School of Data Sciences, Zhejiang University of Finance and Economics, 310018 Hangzhou, China

[*] Corresponding Author: Trung Nghia Vu, TrungNghia.Vu@ki.se

Abstract

An individualized cancer therapy is ideally chosen to target the cancer's driving biological pathways, but identifying such pathways is challenging because of their underlying heterogeneity and there is no guarantee that they are druggable. We hypothesize that a cancer with an activated druggable cancer-specific pathway (CSP) is more likely to respond to the relevant drug. In this study we develop and validate a systematic method to search for such CSPs, by (i) introducing a pathway activation score (PAS) that integrates cancer-specific driver mutations and gene expression profile, and drug-specific gene targets; (ii) applying the method to identify CSPs from pan-cancer datasets; (iii) analysing the correlation between PAS and the response to relevant drugs. In total, 4,794 CSPs from 23 different cancers are discovered in the Genomics of Drug Sensitivity in Cancer database and validated in The Cancer Genome Atlas database. Supporting the hypothesis, for the CSPs in acute myeloid leukemia, cancers with higher PASs are shown to have stronger drug response, and this is validated in the BeatAML cohort. All CSPs are publicly available at https://www.meb.ki.se/shiny/truvu/CSP/.

Key words: cancer-specific pathways; pathway activation score; AML

Introduction

Cancer is the second leading cause of deaths and was responsible for 9.6 million deaths worldwide in 2018. Approximately, one in six deaths is due to cancer [1]. Cancer can result from an uncontrollable cell growth due to genetic alterations in their genomes [2] that change the biological function of some oncogenes and their associated pathways. Drugs designed for specific gene targets may not work as expected in a specific cancer because of the underlying heterogeneity in its driving biological pathways. To kill a specific cancer with an inhibitor, theoretically we need to find one that can down-regulate the cancer's driving pathway(s). There are at least two immediate challenges: (i) Pathway activation is only a necessary but not sufficient condition for its driving property and empirically we can observe many activated pathways in any given cancer, so it is not obvious how to determine which is the driving pathway; (ii) The driving pathway may not have druggable targets, for example, the driving pathway has a poor functional connectivity with the targets of the drug, leading to no impact of the drug on the driving pathway. Thus in our approach a pathway activity is first measured by the mRNA expression of the genes in the pathway. The pathway activity is weighted by the functional connectivity between the pathway, potential driver genes and drug targets. Then, we search for pathways that are uniquely activated in specific cancers but not in others. We focus on druggable pathways, roughly those have known drug targets. (In the actual computation we also allow genes upstream to the targets.) We hypothesize that a cancer with an activated druggable cancer-specific pathway (CSP) is more likely to respond to the relevant drug. Thus our aim in this study is to develop and validate a systematic method to search for such CSPs.

Many studies [3, 4, 5] have investigated universal cancer signaling pathways. For instance, the p53, RTK-RAS signaling or cell cycle pathways are frequently altered across different cancers [6]. Recently, Sanchez and colleagues [7] analysed the mechanisms and patterns of somatic alterations in 10 common canonical pathways in different cancers using The Cancer Genome Atlas (TCGA) cohort: cell cycle, Hippo, Myc, Notch, Nrf2, PI-3-

Compiled on: July 15, 2022.
Draft manuscript prepared by the author.

Figure 1. Overview of identifying CSPs from the pharmacogenomics data. Panel A: Pathway activation score (PAS) is computed from the pharmacogenomics data of GDSC along with pathway and drug target databases. In the illustration of PAS, Tanespimycin or 17-AAG has a target gene HSP90 which involves in pathway PI3k/AKT. For simplicity, the full information of the pathway is not shown in this example. The main analysis includes: 1) identification of CSPs from the GDSC cohort with validation using the TCGA cohort (panels B and C); and 2) investigation of the association between PAS and drug responses with validation using the BeatAML cohort (panels D and E). These plots are derived from the analyses of the PASs of Martens-PML-RARA [20] druggable by quizartinib in Acute Myeloid Leukemia (AML). The boxplots (panels B and C) show that the PAS of AML is over-expressed while the PASs of other cancers are low-expressed and not significantly different from each other. Panels D and E: each point presents a tumor, and the lines are linear-regression lines. The values of PAS and AUC in the plots are under the normal score transformation, see the Material and Method section.

Kinase/Akt, RTK-RAS, TGFβ signaling, p53, and β-catenin/Wnt. However, some altered signaling pathways appear limited to specific tumors; for example, some pathways of BRCA1 and BRCA2 tumour-suppressor genes are known to be specific to breast and ovarian cancers [8, 9, 10]. Altered signaling pathway due to the chromosomal rearrangement event of PML-RARA fusion [11] is often observed only in acute promyelocytic leukemia (APL), a distinct subgroup of acute myeloid leukemia (AML). Here we shall consider only pathways that are cancer specific.

For a given altered signaling pathway that is specific to a cancer, different drugs can affect the pathway differently, thereby potentially producing distinct levels of drug-response. Conceptually we expect the action a drug from the role of its targets in the pathway. For instance, midostaurin and gilteritinib are inhibitors that target mutations of a type III receptor—tyrosine kinase (FLT3) [12], which occur in 30% of AML cases [13]. So, the action of these inhibitors should be assessed in activated pathways that contain the FLT3 gene. Therefore, the investigation of a signaling pathway specific to a cancer is more informative clinically if it mediates the action of a specific drug. In other words, the pathway is druggable, so we need to capture the element of druggability in the definition of the pathway activity.

In this study, we develop a systematic methodology to identify and validate druggable cancer-specific pathways. Briefly, we compute pathway activation score (PAS) to represent the activity level of pathways for specific cancers and take drug targets into account. The PAS of a tumor is calculated for each (drug, pathway) pair using information of gene expression and driver genes of the tumor and target genes of the drug. Then, we implement cancer-specific analysis to discover the cancer-specific pathways (CSPs) that exhibit high activation only in one single cancer while activation scores of the pathways in other cancers are not significantly different from each other. The workflow of the study is present in Figure 1. First, we apply the proposed method to identify CSPs from the Genomics of Drug Sensitivity in Cancer (GDSC) cohort [14] as the discovery set, which contain 23 different cancers and 251 drugs. Then, the CSPs are validated in the TCGA cohort [15]. Finally, utilizing the fruitful omic and drug data of BeatAML study [16], we will focus on the CSPs of AML, the most common type of leukemia cancer in adult with high relapse rate (50% within 6 months) and poor survival outcome (only 10% within 5 years) [17, 18]. Some recent studies also consider the integration of the GDSC and the BeatAML cohort. For example, Jafari et al. uses the drug data from two cohorts to develop bipartite network models to search for combination therapies in AML [19]. In support of our hypothesis, for the CSPs in acute myeloid leukemia, cancers with higher PASs are shown to have stronger drug response, and this is validated in the BeatAML cohort.

Results

Pathway activation score

PAS is defined as a tumor-specific pathway activity level that is relevant to a specific drug. It is calculated based on the connection between the driver gene(s), the drug-specific target gene(s) and the tumor-specific mRNA expression level of the genes in the pathway. In this study, PAS of a tumor is calculated for each (drug, pathway) pair. Genes in a pathway P are classified into two groups: (i) G_u, which includes both the target and upstream genes, and (ii) G_d, which contains the downstream genes. We first compute an upstream activity score $S(G_u)$ as the sum of mRNA expression of the genes in G_u. Next, the score is weighted by the functional network connectivity between the gene sets of the driver genes, the target genes and the pathways using the network enrichment analysis (NEA) [21], which is described in further details in the Materials and Methods section. Three connectivity weights w_1, w_2, and w_3 are computed for these pairs of gene sets: (driver genes ↔ target genes), (driver genes ↔ pathway gene sets) and (target genes ↔ pathway gene sets). Each weight ranges from zero to one, where zero indicates little or no functional interaction and one indicates a high interaction. The final PAS_u is calculated as $S(G_u) * (1 + w_1 + w_2 + w_3)$. In the implementation, we identify recurrent mutations and fusions in each tumor as the potential driver genes; more details are given in the Materials and Methods section. The pathway score for downstream activity PAS_d is computed similarly. Figure 1A illustrates a toy example of PAS for PI3k/ATK pathway targeted by tanespimycin.

For the purpose of identifying CSPs we need to define a scalar PAS. Our hypothesis is that for a drug to be effective on a tumor, its target genes should be part of a pathway that is highly activated, where high activation is measured relative to the other part of the pathway. So we focus on the positive PAS $\equiv \text{PAS}_u - \text{PAS}_d$ as the primary pathway activation score. To further support the hypothesis, we also need evidence that the downstream activation is not informative of drug response. As a motivation, suppose the driver is downstream of the target and that part of the pathway (PAS_d) is highly activated, while the target (or PAS_u) is not activated. Then, intuitively, in this case an inhibitor will not have a chance to work. This is the case where $\text{PAS}_d - \text{PAS}_u > 0$, where the downstream activation is measured relative to the upstream activation. So we also investigate this secondary version of PAS as a measure of version downstream activation and expect no correlation with drug response.

PAS is computed for a set of biological pathways $P = P_1, .., P_N$, a set of drugs $D = D_1, .., D_M$, and a set of tumor samples $S = S_1, .., S_K$ from Z types of cancers $C = C_1, .., C_Z$. A PAS of tumor sample S_k, drug D_i, and pathway P_j is PAS(S_k, D_i, P_j), or simply PAS if it is clear from the context. Thus, given tumor S_k, PAS is calculated for each (D_i, P_j) pair. In practice we use $N = 4,762$ curated human pathways from the MSigDB database. Using the GDSC data as the discovery set, we have $M = 251$ drugs, and $K = 684$ samples from $Z = 23$ cancer types. The target genes of drugs are provided from the GDSC cohort and extended with the curated information from the DrugBank database [22]. The direction of regulatory interactions between genes is taken from multiple directed network databases including HTRIdb [23], regulatory target gene sets of the MSigDB database [24], transcriptional-factor target database of UCSC Genome Browser Database [25], and kinase-substrate interaction database [26].

Identification of cancer-specific pathways

Figure 1 presents an overview of the process used in this study to identify CSPs. First, the gene expression data from GDSC are obtained to calculate PAS. The list of the cancers, their abbreviation and number of samples of each cancer are provided in Table S1. Next, CSP analysis is applied to discover CSPs based on PASs. The

Figure 2. A. The number of CSPs identified in the GDSC cohort and the TCGA cohort. For each cancer in the x-axis, the left-most (blue) barplot represents the results of the GDSC cohort, the middle (orange) barplot shows the number of CSPs of the TCGA cohort, and the right-most (red) barplot is the number of validated CSPs. The y-axis is presented in log2 scale and the cancers in the x-axis are ordered by their number of validated CSPs. **B** and **C.** The rediscovery rate (RDR) of CSPs in terms of the association between PAS and drug sensitivity in AML. RDR is the proportion of the top 5%, 10%, 20%, 30%, 40%, 50%, 100% CSPs identified in the discovery set (GDSC cohort) that is significant in the validation set (BeatAML cohort). (B) RDR of CSPs with negative correlations and (C) RDR of CSPs with positive correlations.The horizontal dashed lines present the target lines for the target levels of p-value at $\alpha = 0.05$ (red) and 0.01 (blue). **D.** PAS of Martens-PML-RARA (P_j) druggable by quizartinib (D_i) from the GDSC cohort in comparison with PASs of following three groups: 1) same pathway but different drugs ($\overline{D_i}, P_j$), 2) same drug but different pathways ($D_i, \overline{P_j}$), and 3) different drugs and different pathways ($\overline{D_i}, \overline{P_j}$). P-values of the permutation test are presented on the top of each pair. $\overline{D_i}$ represents the set of other drugs, while $\overline{P_j}$ refers to the set of other pathways. The values in the parentheses of x-axis are the numbers of samples for each group. The y-axis presents PAS values of the groups.

CSP analysis takes into account all (drug, pathway, cancer) triplets to discover (drug, pathway) pairs which are specific to the cancer. Finally, the CSPs are validated using TCGA cohort. For the CSPs in AML, we assess the association between PAS and drug sensitivity and validate it in the BeatAML cohort. More details are described in the section Materials and Methods.

From a total of 250,479 CSP candidates across 23 cancers and 251 drugs in the GDSC cohort, we identify 69,986 CSPs with t-statistics FDR < 0.01 and those within the first quartile of χ^2-statistics. Figure S1A displays the distributions of the statistics of these CSPs. Among these cancers, colon/rectum adenocarcinoma (COAD/READ) has the largest number of CSPs (17,057; 24,37%), followed by breast cancer (BRCA), skin cutaneous melanoma (SKCM), and pancreatic adenocarcinoma (PAAD) more than 4,000 (> 5%) CSPs (see Figure 2A). In contrast, some cancers report only few CSPs, for example, 112 and 233 for stomach adenocarcinoma (STAD) and thyroid carcinoma (THCA), respectively. Details of the numbers and proportions of CSPs identified in individual cancers are provided in Table S2 and Figure S2. All CSPs are available at `https://www.meb.ki.se/shiny/truvu/CSP/`.

Validation of CSPs in the TCGA cohort

Using the same computational procedure, 4,794 CSPs discovered in the GDSC cohort are validated in the TCGA cohort. Figure 2A shows the number of validated CSPs for each cancer using the TCGA cohort; details are mentioned in Table S2. BRCA has the largest number of validated CSPs (1,284), followed by AML (992). However, the validation rate of BRCA is relatively low (0.16) in comparison to AML (0.33), prostate adenocarcinoma (PRAD) (0.51), and ovarian cystadenocarcinoma (OV) (0.53). The numbers of validated CSPs of PRAD (284) and OV (160) are about five times less than the one of BRCA. These diseases also have the top validation rates, while the other diseases have a small validation proportion of less than 20%. The number of validated CSPs and the validation rate of individual cancers are provided in Table S2. The number of CSPs found in the TCGA cohort (n = 110,400) is higher than that in the GDSC cohort (n = 69,986). There are several possible reasons for the difference: the GDSC cohort contains data from cell lines, which tend to be more homogeneous compared to the patient-derived data from the TCGA cohort. Furthermore, the number of samples of each cancer in the TCGA cohort are much higher than those in the GDSC cohort (Supplementary Table S1), which increases the sensitivity in the test of differences.

Correlation between PAS and drug sensitivity

Next we investigate the correlation between PAS and drug sensitivity in AML, the disease with a high validation rate and for which there exist extensive drug response assays in multiple datasets. Drug sensitivity is measured in terms of area under the curve (AUC) of cancer-cell survival as a function of drug dose. A small AUC indicates a good drug response, i.e. the drug kills the cancer cells at the low end of the dose range. A negative correlation cor(PAS, AUC) means high PAS is associated with better drug response. This happens if the drug is effective in killing the cancer cells and the pathway *P* mediates the drug response. Such an observation would support our main hypothesis that cancers with activated druggable CSPs are likely more responsive to the relevant drug. If there is no correlation, it is either because the drug is not effective, e.g., there is drug resistance, or because its effect is mediated by other pathways. A positive correlation means that higher PAS is associated with worse drug response, or lower PAS with better response, which is opposite to our hypothesis. As we do not expect positive correlation, this part can be used as a negative control. Further details are in the Materials and Methods. Data from the BeatAML cohort are used for validation. Figures 1D andE present an example of CSPs with the involvement of drug quizartinib, pathway Martens-PML-RARA [20], and AML, where the correlation between PAS and AUC is −0.14 in the GDSC cohort and −0.20 in the BeatAML cohort. From all identified CSPs for AML in the GDSC cohort, we collect 1,007 CSPs that share 56 overlapped drugs with the BeatAML cohort. PASs of these CSPs are also calculated in the BeatAML cohort. The CSPs are first ranked by the correlation cor(PAS, AUC) in the GDSC. This rank is also used later for the results showed in Figure 2. We assess the validation by computing the rediscovery rate (RDR), defined as the proportion of the top-ranking CSPs identified in GDSC that have significant cor(PAS, AUC) in the validation set (BeatAML). CSPs with p-value $< \alpha$ are considered as significant, using target levels $\alpha = 0.05$ and 0.01.

Figure 2B presents the RDRs of the set of CSPs with negative correlations. Here, the x-axis represents 5%, 10%, 20%, 30%, 40%, 50%, 100% top-ranking CSPs in the discovery set (GDSC); the y-axis represents the corresponding RDRs at 0.05 (red line) and 0.01 (blue line) thresholds. Both RDR curves generally slope downwards when the number of top CSPs increases and closely reaches to the target (horizontal dashed lines) at top 100% (the full set). From top 5% to top 20% of the red line, RDRs archive the highest value at $\sim$ 0.20. Table S3 shows 28 CSPs at top 20% that are rediscovered in the validation set. Our analyses of the cor(PAS_u, AUC) and the cor(PAS_d, AUC) of these 28 CSPs show that the downstream pathway activation should be uninformative towards drug response (data not shown). Figure 2C presents the RDRs for the set of CSPs with positive correlations. The results show that most RDRs are close to the target lines (the horizontal dashed lines in the figure), supporting our expectation that there are no CSPs where lower PAS is associated with better drug response.

Figure 1B (with extension in Figure S3A) illustrates PASs of a top CSP (ranked based on t-statistics) of AML versus other cancers in the GDSC cohort. This AML-specific CSP is the Martens_bound_by_PML_RARA_fusion (Martens-PML-RARA). which is druggable by quizartinib. Median PAS of AML (24.3) is 2.5 times greater than that of the remaining cancers (median = 9.7). The pattern is validated in the TCGA cohort (see Figure 1C with extension in Figure S3B). The pathway was first described by Martens and colleagues [20] in the study on genes with promoters occupied by PML-RARA fusion in acute promyelocytic leukemia (APL), a well-studied subtype of AML disease [27]. Intriguingly, quizartinib is a small molecule receptor tyrosine kinase inhibitor that targets to FLT3 genes and has been shown to work for FLT3-mutated AML cases [28]. The FLT3 mutation is the one of the most common mutations in AML which can be caused by the internal

tandem duplication of FLT3 (FLT3-ITD), point mutations, and indels in the tyrosine kinase domain (FLT3-TKD)[29]. Among APL patients, 47.9% carries FLT3 mutations [30], and it has been shown that PML-RARA fusion can collaborate with FLT3 mutation to induce an APL-like disease in the mouse [31].

We then investigate the correlation between the downstream activation and the drug sensitivity. Here PAS is defined as PAS_d - PAS_u so a high positive PAS corresponds to the downstream part of the pathway having higher activation relative to the upstream part. A similar procedure is applied for this version of PAS to compute the RDRs. Supplementary Figures S4A and B show the RDRs of the set of CSPs with negative and positive correlations, respectively. The RDRs generally follow the P-value target lines (0.05 and 0.01) closely, indicating there is no evidence of correlation between downstream activation with drug response.

Specificity of CSPs in AML

We further investigate the specificity of the identified CSPs of AML using the case in Figure 2C as an example. Given D the set of drugs and P the set of pathways from the CSPs identified in AML, we define $\overline{D_i} = \{D_m | D_m \in D, m \neq i\}$ as the set of the other drugs. Similarly, $\overline{P_j}$ is defined as the set of other pathways. Suppose, a CSP is specified by a combination of drug D_i and pathway P_j in AML. Then we investigate the over-expression of its PASs in comparison to PAS these three other sets: 1) the same pathway but different drugs $(\overline{D_i}, P_j)$, 2) the same drugs but different pathways $(D_i, \overline{P_j})$, and 3) different drugs and different pathways $(\overline{D_i}, \overline{P_j})$. To compare the PASs of group (D_i, P_j) with another group, we use a permutation test where the null distribution of the t-statistic is generated by random permutation of cell-line labels. A total of 10,000 permutations are carried out to build the null distribution. Then, the actual t-statistic and the population of the t-statistics from permuted dataset are used to calculate the empirical p-values.

Figure 2D presents the results of permutation test for quizartinib and Martens-PML-RARA combination [20] from the GDSC cohort. The results show that PASs of this CSP (group $[D_i, P_j]$) are significantly higher than that of the groups of different pathways or both drugs and pathways $(D_i, \overline{P_j})$ and $[\overline{D_i}, \overline{P_j}]$; p-value = 1e-4), indicating that quizartinib is more closely linked to the Martens-PML-RARA pathway compared to the other pathways. Compared to the group of the same pathway but different drugs, this CSP also has significantly higher PASs (p-value < 1e-4). Similar results are also observed for the other CSPs of AML. The details are provided in Table S4 and illustrated in the interactive website.

Discussion and Conclusion

To investigate the hypothesis that cancers with activated druggable CSPs are more likely to respond to the relevant drugs, we have introduced PAS and apply it to conduct a systematic search of druggable cancer-specific pathways in 23 cancers from the GDSC cohort. The CSPs of these cancers are then validated in the TCGA cohort. In support of the hypothesis, we observe a significant correlation between higher PAS and stronger drug response among the CSPs identified in AML and validate this in the BeatAML cohort. All results are provided in an interactive website available to users.

PAS is defined to capture the druggability of a pathway for an individual cancer. In principle, this information can be used to build a model for predicting drug responses of tumors in precision medicine. Current models often apply black-box statistical and machine learning methods to multiple omics data to predict responses of a single drug (monotherapy) or combination of drugs (drug synergy) [32, 33]. This sometimes makes the interpretation of the prediction models difficult [34]. One of the advantages of using PASs for the prediction model is its ability to keep track of the driving mechanisms through the pathway information. Furthermore, PASs can be applied to prediction in both monotherapy or drug synergy as long as the target gene list is collected from the drug(s).

This study is also conducted using the rich resources; however, the data still have some weaknesses. Firstly, information on drug target genes is often incomplete, and off-target genes are generally unknown. We collect the target gene list provided from the GDSC cohort and extend with the curated information from the DrugBank database [22]. Recently, a community effort has been made to improve the target space of drugs via a web platform named Drug Target Commons [35]. Investigating the use of the drug target data of this database will be our future work. Secondly, the pathway databases are still incomplete, and we expect they would be improved in the future. Thirdly, the number of cell lines of individual cancer in GDSC is limited and could not be the representative for the real data of the disease. Fourthly, the GDSC and BeatAML cohorts only share a small number of drugs; this means, a large number of CSPs are not assessed in terms of drug response. This problem can be improved by producing more drug data. Despite the limited sharing drugs, integration of the two cohorts is considered in some recent studies. For example, Jafari and colleagues propose bipartite network models to search for combination therapies in AML using the data from both GDSC cohort and BeatAML cohort [19]. Finally, there is general lack of publicly available drug data of other cancers for validation.

Materials and Methods

Functional network connectivity between driver genes, pathway and target genes of drugs

To achieve the weights for PAS using the interaction between driver genes, pathway and drug-target genes, we utilize the network enrichment analysis (NEA) [21]. Briefly, NEA originally assesses the functional network connectivity between two gene sets: a functional gene set (FGS), e.g., driver alteration and an altered gene sets (AGS) associated with a certain downstream biological state, e.g., differentially expressed (DE) genes. Comparing to the traditionally used gene-set enrichment analyses (GSEA) [36], NEA extends GSEA with topological information in terms of gene interaction networks which provide biologically informative category. A comprehensive network contains 1,445,027 functional links between 16,299 distinct HUPO genes is considered in the analysis.

In application to this study, NEA is applied for three pairs of gene sets including driver genes, pathway genes and drug-target genes. For each pair, one gene set is selected for FGS and the remaining gene set is for the AGS. In particular, FGS is assigned for the set of drug-target genes in (drug-target genes, pathway genes) and (drug-target genes, driver genes) while for (driver genes, pathway), the driver genes are used for FGS. Finally, NEA simplifies the assessment of the functional connectivity by defining an enrichment score as:

$$z = \frac{d_{AF} - \overline{d}_{AF}}{\sigma_{AF}} \quad (1)$$

where d_{AF} is the number of connected link between AGS and FGS; $\overline{d}_{AF}$ and σ_{AF} are the mean and standard deviation of d_{AF} respectively, which are estimated on a randomize network under the null hypothesis. Thus, for each PAS, we collect three corresponding z-scores expressing the over-representation of drug-target genes on cancer driver genes (z_1), driver genes on pathway genes (z_2), and target genes on pathway genes (z_3) based on the functional gene network. Finally, these three enrichment scores are then converted into normal probability scores (w_1, w_2, and w_3) which are used as

the weights for PAS.

Discoveries of cancer-specific pathways

Given a drug D_i, a pathway P_j is considered as specific to a cancer C_z, that is, CSP, if the pathway over-activates in that cancer while activation scores of this pathway in other cancers are not significantly different from each other (see Figure 1B). The issue is straightforward: If we consider only two cancers, a standard statistical approach such as t-test can be applied directly to PASs. However, when there are more than two cancers, for example, 23 different cancers from GDSC cohort (as in this study), the standard method only ensures that a cancer is different from the rest, but the remaining cancers might be different from each other. Therefore, in this case, the specificity of the pathway for the remaining cancers is not guaranteed. To identify the CSPs, we apply a two-statistic approach originally developed in a recent study [37] for the PAS data of GDSC cohort. The method provides two statistics for each cancer: 1) a robust t-test (T_1) for comparing between that cancer and the rest, and 2) a χ^2-statistic (T_2) for jointly comparing the remaining cancers.

For an activated pathway to cancer specific, we expect a large t-statistic for T_1 and a small χ^2-statistic for T_2. To account for multiple testing, the false discovery rates (FDRs) [38] of T_1 are calculated, and we keep CSPs with FDR < 0.01. We further keep only CSPs whose χ^2-statistics are within the first quartile. Finally, we apply the following sample size conditions: 1) For each CSP, the number of samples for each supporting cancer is larger than five, and 2) it is supported by at least three cancers.

Pathway activation score in relation to drug response

Our hypothesis is supported if the pathway P_j mediates the response to drug D_i in cancer C_z; statistically this is the case if the pathway activity of CSP(D_i, P_j, C_z) correlates with the drug response. Figure 1D shows an example in AML of the relation between PAS and the area under curve (AUC) of drug sensitivity of the pathway Martens-PML-RARA [20] druggable by quizartinib, where the AUCs are obtained from cell lines actually treated with quizartinib. Given a CSP(D_i, P_j, C_z), we first apply the normal score transformation on both PAS and drug sensitivity (AUC) of the tumors in cancer C_z. Subsequently, we calculate the Pearson correlation between PAS and AUC as cor(PAS, AUC). Here, two versions of PAS for upstream and downstream activation are used to compute cor(PAS, AUC). The PAS of the upstream version is defined as PAS = PAS$_u$ - PAS$_d$, while for the downstream version PAS = PAS$_d$ - PAS$_u$. As activation, only positive values are considered.

Datasets

This study uses the data of GDSC cohort as the discovery set. Validation sets have been obtained from the following sources: 1) TCGA cohort, 2) Therapeutically Applicable Research to Generate Effective Treatments (TARGET) cohort, and 3) BeatAML cohort.

GDSC dataset: GDSC project [14] has been undertaken with the aim of discovering cancer biomarkers that are highly responsive to anti-cancer drugs. This cohort contains the genomic information of more than 1000 human cancer cell lines and drug sensitivities of more than 250 drugs.

The drug data of GDSC cohort (version 17.3) contains a total of 224,202 cell line-drug experiments from 251 drugs and 1,065 cell lines. In this study, we use only 125,894 monotherapy profiles of 684 cell lines from 23 cancers after removing the profiles with more than one replicate. The number of cell lines of a cancer ranges from 6 to 64; AML has 28 cell lines. The potential driver genes of the samples including mutations and fusion genes, are collected from Depmap Portal [39]. We keep mutations with occurrence at least 2% of total samples across cancers. For the fusion genes, we keep all fusions with at least 2 occurrences and overlapping with the fusions found in the Mitelman database [40]. The expression data of 17,715 genes from these cell lines are also achieved.

TCGA and TARGET datasets: TCGA [15] is led by the National Cancer Institute's Center for Cancer Genomics and the National Human Genome Research Institute with the aim of providing a landscape of genomic characterization for more than 33 malignant diseases. TARGET is an ongoing-project that provides the comprehensive genomic landscape targeted toward countering childhood cancer. In validation step, we collect data of 22 cancers from TCGA cohort and neuroblastoma [NB] from TARGET cohort [41]. These cancers are matched with the cancers in the GDSC cohort of the discovery set. The data contain expressions of 37,636 genes from a total of 8,825 samples across 23 cancers. The detailed information of these cancers is provided in Supplementary Table S1. Gene expressions normalized by Fragments Per Kilobase of transcript per Million mapped reads (FPKM) originally reported from the cohorts are converted to Transcript per Million (TPM) for downstream analyses. Mutations and fusion genes are also collected and filtered to obtain potential driver genes with high occurrence. Frequent mutations with occurrence at least 1% of total samples are kept, and the same filter in the GDSC cohort is applied for fusion genes.

BeatAML dataset: BeatAML [16] is an ongoing project that aims to provide an extensive landscape of AML, comprising clinical, genomic, and drug response data. This cohort contains RNA-seq samples of 461 AML cases. These samples are sequenced by the Illumina HiSeq 2500 platform (100bp paired-end reads) after processing with Agilent SureSelect Strand-Specific RNA Library Preparation Kit on the Bravo robot. The FASTQ files of these samples are input to XAEM [42]; then expressions in transcripts per millions (TPM) of 26,086 genes are collected. After removing unexpressed genes ($TPM \leq 1e-2$ in more than 90% of samples), 23,035 genes remain. The mutations and fusion genes collected from the BeatAML cohort are used. The fusion genes are filtered by the same procedure in the GDSC cohort. The drug sensitivities of 122 compounds reported in terms of both IC_{50} and AUC are also collected. The data consist of 47,650 records from 528 AML patients.

The results of this study are available at https://www.meb.ki.se/shiny/truvu/CSP/.

Data availability

The implementations of PAS generation and the shiny application are available at https://github.com/tracquangthinh/CSP. All related datasets can be downloaded from a public Zenodo repository at https://doi.org/10.5281/zenodo.6787033.

Acknowledgements

This work was partially supported by funding from the KI Research Foundation, the Swedish Research Council (VR) and the Swedish Foundation for Strategic Research (SSF). The computations were enabled by resources provided by the Swedish National Infrastructure for Computing (SNIC) in Uppsala, which is partially funded by the Swedish Research Council through grant agreement no. 2018-05973.

Author contributions

TNV and YP initiated and oversaw the study. QTT, TNV and YP contributed to method development and manuscript writing. QTT, TZ and TNV performed the bioinformatics analysis and webpage

development with input from YP.

Competing interests

The authors declare no competing interests.

References

1. WHO, Latest global cancer data: Cancer burden rises to 18.1 million new cases and 9.6 million cancer deaths in 2018; 2018. https://www.who.int/cancer/PRGlobocanFinal.pdf.
2. Cooper GM. The Cell. 2nd ed. Sunderland (MA): Sinauer Associates; 2000.
3. McLendon R, Friedman A, Bigner D, Van Meir EG, Brat DJ, M Mastrogianakis et al G. Comprehensive genomic characterization defines human glioblastoma genes and core pathways. Nature 2008;455(7216):1061–1068. https://www.nature.com/articles/nature07385.
4. Ding L, Getz G, Wheeler DA, Mardis ER, McLellan MD, Cibulskis et al K. Somatic mutations affect key pathways in lung adenocarcinoma. Nature 2008;455(7216):1069–1075. https://www.nature.com/articles/nature07423.
5. Jones S, Zhang X, Parsons DW, Lin JCH, Leary RJ, Angenendt et al P. Core Signaling Pathways in Human Pancreatic Cancers Revealed by Global Genomic Analyses. Science 2008;321(5897):1801–1806. https://science.sciencemag.org/content/321/5897/1801.
6. Vazquez A, Bond EE, Levine AJ, Bond GL. The genetics of the p53 pathway, apoptosis and cancer therapy. Nature Reviews Drug Discovery 2008;7(12):979–987. https://www.nature.com/articles/nrd2656.
7. Sanchez-Vega F, Mina M, Armenia J, Chatila WK, Luna A, La et al KC. Oncogenic Signaling Pathways in The Cancer Genome Atlas. Cell 2018;173(2):321–337. https://www.cell.com/cell/abstract/S0092-8674(18)30359-3.
8. Roy R, Chun J, Powell SN. BRCA1 and BRCA2: different roles in a common pathway of genome protection. Nature Reviews Cancer 2012;12(1):68–78. https://www.nature.com/articles/nrc3181.
9. Hill SJ, Clark AP, Silver DP, Livingston DM. BRCA1 Pathway Function in Basal-Like Breast Cancer Cells. Molecular and Cellular Biology 2014;34(20):3828–3842. https://mcb.asm.org/content/34/20/3828.
10. Welcsh PL, King MC. BRCA1 and BRCA2 and the genetics of breast and ovarian cancer. Human Molecular Genetics 2001;10(7):705–713. https://doi.org/10.1093/hmg/10.7.705.
11. Casorelli I, Tenedini E, Tagliafico E, Blasi MF, Giuliani A, Crescenzi et al M. Identification of a molecular signature for leukemic promyelocytes and their normal counterparts: focus on DNA repair genes. Leukemia 2006;20(11):1978–1988. https://www.nature.com/articles/2404376.
12. Luger SM, Sun Z, Loghavi S, Lazarus HM, Rowe JM, Tallman et al MS. Phase II Randomized Trial of Gilteritinib Vs Midostaurin in Newly Diagnosed FLT3 Mutated Acute Myeloid Leukemia (AML). Blood 2019;134:1309–1309. https://doi.org/10.1182/blood-2019-128377.
13. Carter JL, Hege K, Yang J, Kalpage HA, Su Y, Edwards et al H. Targeting multiple signaling pathways: the new approach to acute myeloid leukemia therapy. Signal Transduction and Targeted Therapy 2020;5(1). https://www.nature.com/articles/s41392-020-00361-x.
14. Yang W, Soares J, Greninger P, Edelman EJ, Lightfoot H, Forbes et al S. Genomics of Drug Sensitivity in Cancer (GDSC): a resource for therapeutic biomarker discovery in cancer cells. Nucleic Acids Research 2013;41:955–961. https://doi.org/10.1093/nar/gks1111.
15. Weinstein JN, Collisson EA, Mills GB, Shaw KRM, Ozenberger BA, Ellrott et al K. The Cancer Genome Atlas Pan-Cancer analysis project. Nature Genetics 2013;45(10):1113–1120. https://www.nature.com/articles/ng.2764.
16. Tyner JW, Tognon CE, Bottomly D, Wilmot B, Kurtz SE, Savage et al SL. Functional genomic landscape of acute myeloid leukaemia. Nature 2018;562(7728):526–531. https://www.nature.com/articles/s41586-018-0623-z.
17. Felicitas T, Arnold G. Treatment of Relapsed Acute Myeloid Leukemia. Current treatment options in oncology 2020;21(8):66.
18. Ganzel C, Zhuoxin S, Larry DC, Hugo FF, Dan D, Jacob MRea. Very poor long-term survival in past and more recent studies for relapsed AML patients: the ECOG-ACRIN experience. American journal of hematology 2018;93(8):1074–1081.
19. Jafari M, Mehdi M, Jie B, Farnaz B, Shuyu Z, Johanna Eea. Bipartite network models to design combination therapies in acute myeloid leukaemia. Nature communications 2022;13(1):1–12.
20. Martens JH, Brinkman AB, Simmer F, Francoijs KJ, Nebbioso A, Ferrara F, et al. PML-RARα/RXR alters the epigenetic landscape in acute promyelocytic leukemia. Cancer cell 2010;17(2):173–185.
21. Alexeyenko A, Lee W, Pernemalm M, Guegan J, Dessen P, Lazar et al V. Network enrichment analysis: extension of gene-set enrichment analysis to gene networks. BMC bioinformatics 2012;13(1):1–11.
22. Wishart DS, Feunang YD, Guo AC, Lo EJ, Marcu A, Grant JRea. DrugBank 5.0: a major update to the DrugBank database for 2018. Nucleic Acids Research 2018;46:1074–1082.
23. Bovolenta LA, Acencio ML, Lemke N. HTRIdb: an open-access database for experimentally verified human transcriptional regulation interactions. BMC Genomics 2012;13(1):405. https://doi.org/10.1186/1471-2164-13-405.
24. Liberzon A, Birger C, Thorvaldsdóttir H, Ghandi M, Mesirov JP, Tamayo P. The Molecular Signatures Database Hallmark Gene Set Collection. Cell Systems 2015;1(6):417–425. https://www.cell.com/cell-systems/abstract/S2405-4712(15)00218-5.
25. Karolchik D, Hinrichs AS, Furey TS, Roskin KM, Sugnet CW, Haussler et al D. The UCSC Table Browser data retrieval tool. Nucleic Acids Research 2004;32:493–496. https://doi.org/10.1093/nar/gkh103.
26. Hornbeck PV, Chabra I, Kornhauser JM, Skrzypek E, Zhang B. PhosphoSite: A bioinformatics resource dedicated to physiological protein phosphorylation. Proteomics 2004;4(6):1551–1561. https://analyticalsciencejournals.onlinelibrary.wiley.com/doi/abs/10.1002/pmic.200300772.
27. Ryan MM. Acute promyelocytic leukemia: a summary. Journal of the advanced practitioner in oncology 2018;9(2):178.
28. Garcia-Horton A, Yee KW. Quizartinib for the treatment of acute myeloid leukemia. Expert Opinion on Pharmacotherapy 2020;21(17):2077–2090.
29. Kiyoi H, Kawashima N, Ishikawa Y. FLT3 mutations in acute myeloid leukemia: Therapeutic paradigm beyond inhibitor development. Cancer science 2020;111(2):312–322.
30. Schnittger S, Bacher U, Haferlach C, Kern W, Alpermann T, Haferlach T. Clinical impact of FLT3 mutation load in acute promyelocytic leukemia with t (15; 17)/PML-RARA. Haematologica 2011;96(12):1799.
31. Kelly LM, Kutok JL, Williams IR, Boulton CL, Amaral SM, Curley et al DP. PML/RARα and FLT3-ITD induce an APL-like disease in a mouse model. Proceedings of the National Academy of Sciences 2002;99(12):8283–8288.
32. Costello JC, Heiser LM, Georgii E, Gönen M, Menden MP, Wang et al NJ. A community effort to assess and improve drug sensitivity prediction algorithms. Nature Biotechnology 2014;32(12):1202–1212. https://www.nature.com/articles/nbt.2877.

33. Menden MP, Wang D, Mason MJ, Szalai B, Bulusu KC, Guan et al Y. Community assessment to advance computational prediction of cancer drug combinations in a pharmacogenomic screen. Nature Communications 2019;10(1):2674. https://www.nature.com/articles/s41467-019-09799-2.
34. Ali M, Aittokallio T. Machine learning and feature selection for drug response prediction in precision oncology applications. Biophysical Reviews 2019;11(1):31–39. https://doi.org/10.1007/s12551-018-0446-z.
35. Tang J, Tanoli ZuR, Ravikumar B, Alam Z, Rebane A, Vähä-Koskela et al M. Drug Target Commons: A Community Effort to Build a Consensus Knowledge Base for Drug-Target Interactions. Cell Chemical Biology 2018;25(2):224–229. https://www.cell.com/cell-chemical-biology/abstract/S2451-9456(17)30426-9.
36. Subramanian A, Tamayo P, Mootha VK, Mukherjee S, Ebert BL, Gillette et al MA. Gene set enrichment analysis: a knowledge-based approach for interpreting genome-wide expression profiles. Proceedings of the National Academy of Sciences 2005;102(43):15545–15550.
37. Vu TN, Pramana S, Calza S, Suo C, Lee D, Pawitan Y. Comprehensive landscape of subtype-specific coding and non-coding RNA transcripts in breast cancer. Oncotarget 2016;7(42):68851–68863. https://www.oncotarget.com/article/11998/text/.
38. Pawitan Y, Murthy KRK, Michiels S, Ploner A. Bias in the estimation of false discovery rate in microarray studies. Bioinformatics 2005;21(20):3865–3872. https://doi.org/10.1093/bioinformatics/bti626.
39. DepMap B, DepMap 21Q4 Public; 2021. https://doi.org/10.6084/m9.figshare.16924132.v1.
40. Mitelman F, Johansson B, Mertens F. Mitelman Database of Chromosome Aberrations and Gene Fusions in Cancer; 2022. https://mitelmandatabase.isb-cgc.org.
41. Pugh TJ, Morozova O, Attiyeh EF, Asgharzadeh S, Wei JS, Auclair et al D. The genetic landscape of high-risk neuroblastoma. Nature Genetics 2013;45(3):279–284. https://www.nature.com/articles/ng.2529.
42. Deng W, Mou T, Kalari KR, Niu N, Wang L, Pawitan Y, et al. Alternating EM algorithm for a bilinear model in isoform quantification from RNA-seq data. Bioinformatics 2020;36(3):805–812. https://doi.org/10.1093/bioinformatics/btz640.

Figure 1

Click here to access/download;Figure;Figure1.pdf

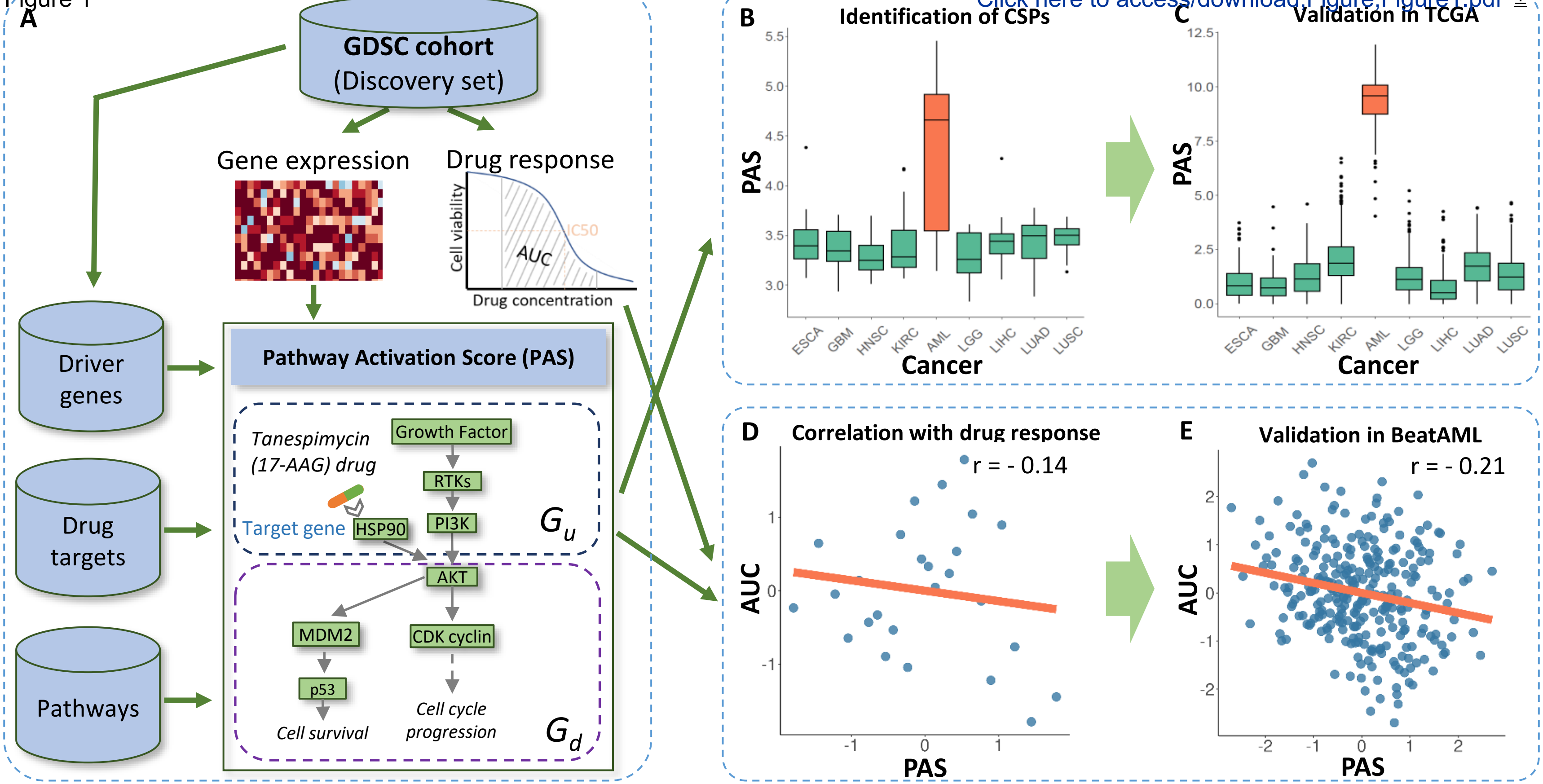

Figure 2

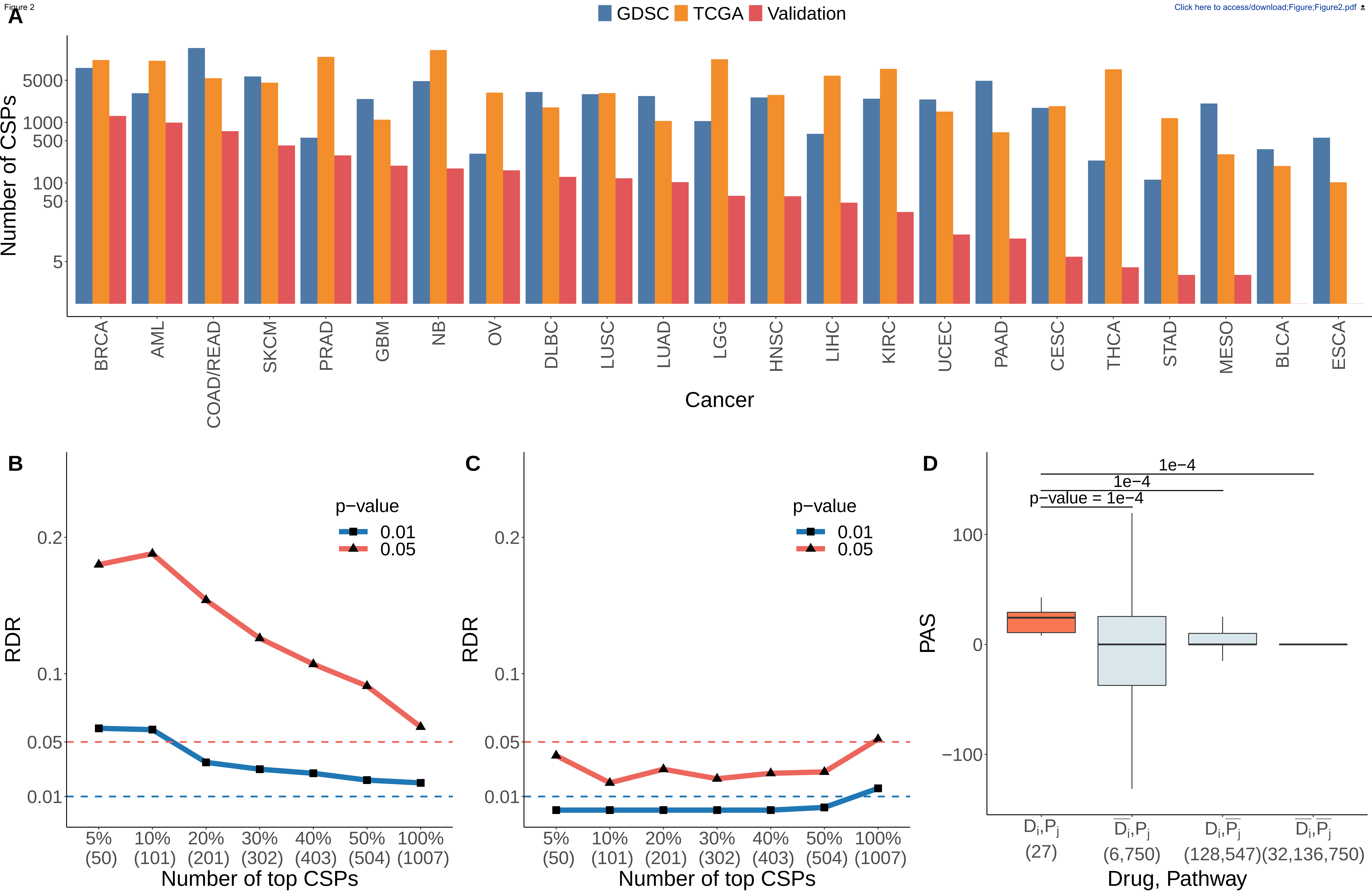
A
GDSC
TCGA
Validation
Number of CSPs
5000
1000
500
100
50
5
BRCA
AML
COAD/READ
SKCM
PRAD
GBM
NB
OV
DLBC
LUSC
LUAD
LGG
HNSC
LIHC
KIRC
UCEC
PAAD
CESC
THCA
STAD
MESO
BLCA
ESCA
Cancer
B
p−value
0.01
0.05
RDR
0.2
0.1
0.05
0.01
5% (50)
10% (101)
20% (201)
30% (302)
40% (403)
50% (504)
100% (1007)
Number of top CSPs
C
p−value
0.01
0.05
RDR
0.2
0.1
0.05
0.01
5% (50)
10% (101)
20% (201)
30% (302)
40% (403)
50% (504)
100% (1007)
Number of top CSPs
D
1e−4
1e−4
p−value = 1e−4
PAS
100
0
−100
Di,Pj (27)
Di,Pj (6,750)
Di,Pj (128,547)
Di,Pj (32,136,750)
Drug, Pathway

Supplementary document

Click here to access/download
Supplementary Material
csp_gigascience_supp.pdf

Personal Cover Click here to access/download;Personal Cover;cover_letter_response_14July2022.docx

Dear Editors,

We are pleased to submit the revision of our manuscript titled "Discovery of Druggable Cancer-Specific Pathways with Application in Acute Myeloid Leukemia" to GigaScience. We thank the reviewers for the constructive comments and suggestions that have improved our study. We have addressed carefully all comments/concerns of the reviewers as described in the attached point-by-point response letter; all the changes are marked in red. Since the responses contain some figures which are not able to include in the "Response to reviewer" box of the submission system, the response letter is concatenated with the main text in a single file. In particular, we have:

- Performed a new analysis of the pathway activation downstream to the drug targets. This is in response to the first major comment from Reviewer 1. We found no evidence of correlation between downstream activation and drug response. This result confirms what we wrote previously.

- Improved the clarity of the specific terms and statements as pointed out by the reviewers.

- Improved the clarity and reproducibility of the pipeline, which has led to slight changes in some results. These changes, however, do not affect the conclusions of the study.

- Enhanced the reproducibility of the study by uploading the source code to produce (i) all components of Pathway Activation Score (PAS) generation and (ii) all the figures of the manuscript at https://github.com/tracquangthinh/CSP

- Updated the related datasets at the Zenodo repository and the corresponding Zenodo link in section Data availability.

We believed that the revision has improved our manuscript. Thank you for your consideration and we look forward to hearing back from you.

Sincerely yours,

Trung Nghia Vu

on behalf of the authors
