# Supplementary material for: Discovery of druggable cancer-specific pathways with application in acute myeloid leukemia
Source: Gigascience. 2022 Sep 29;11:giac091. doi: 10.1093/gigascience/giac091 (PMC9520771; doi:10.1093/gigascience/giac091)
Supplement: giac091_Supplemental_File [file giac091_supplemental_file.pdf]

Supplementary Documents
Discovery of Druggable Cancer-Specific Pathways with Application in Acute Myeloid Leukemia

Quang Thinh Trac et al.

TABLES

Table S1: List of 23 cancers of the GDSC cohort using in this study. Note that COAD and READ in the TCGA cohort are combined together to be consistent with GDSC cohort.

ID	Abbreviation	Name	Cell-lines in GDSC	Samples in TCGA
1	BLCA	Bladder Urothelial Carcinoma	19	414
2	BRCA	Breast invasive carcinoma	51	1109
3	CESC	Cervical squamous cell carcinoma and endocervical adenocarcinoma	14	306
4	COAD/READ	Colon adenocarcinoma/Rectum adenocarcinoma	51	647
5	DLBC	Lymphoid Neoplasm Diffuse Large B-cell Lymphoma	35	48
6	ESCA	Esophageal carcinoma	35	162
7	GBM	Glioblastoma multiforme	36	169
8	HNSC	Head and Neck squamous cell carcinoma	42	502
9	KIRC	Kidney renal clear cell carcinoma	32	539
10	AML	Acute Myeloid Leukemia	28	151
11	LGG	Brain lower grade glioma	17	529
12	LIHC	Liver hepatocellular carcinoma	17	374
13	LUAD	Lung adenocarcinoma	64	535
14	LUSC	Lung squamous cell carcinoma	15	502
15	MESO	Mesothelioma	21	86
16	NB	Neuroblastoma	32	161
17	OV	Ovarian serous cystadenocarcinoma	34	379
18	PAAD	Pancreatic adenocarcinoma	30	178
19	PRAD	Prostate adenocarcinoma	6	499
20	SKCM	Skin Cutaneous Melanoma	55	471
21	STAD	Stomach adenocarcinoma	25	375
22	THCA	Thyroid carcinoma	16	510
23	UCEC	Uterine Corpus Endometrial Carcinoma	9	552

Table S2: The DCSPs discovered from the GDSC cohort and the TCGA cohort across 23 cancers. The validated DCSPs are the DCSPs identified in the GDSC cohort and re-discovered in the TCGA cohort. The last column presents the proportion of validated DCSPs (PV) in the TCGA cohort.

ID	Cancer	DCSPs in GDSC	DCSPs in TCGA	Validated DCSPs	Validation rate	PV in TCGA
1	BLCA	359	190	0	0.00	0.00
2	BRCA	7985	10815	1284	0.16	0.12
3	CESC	1747	1860	5	0.00	0.00
4	COAD/READ	17057	5417	718	0.04	0.13
5	DLBC	3183	1776	125	0.04	0.07
6	ESCA	559	101	0	0.00	0.00
7	GBM	2447	1109	192	0.08	0.17
8	HNSC	2599	2849	59	0.02	0.02
9	KIRC	2470	7727	32	0.01	0.00
10	AML	3049	10515	992	0.33	0.09
11	LGG	1053	11159	60	0.06	0.01
12	LIHC	649	5934	46	0.07	0.01
13	LUAD	2736	1060	102	0.04	0.10
14	LUSC	2937	3054	118	0.04	0.04
15	MESO	2063	296	2	0.00	0.01
16	NB	4826	15753	172	0.04	0.01
17	OV	303	3111	160	0.53	0.05
18	PAAD	4883	687	11	0.00	0.02
19	PRAD	559	12179	284	0.51	0.02
20	SKCM	5772	4555	414	0.07	0.09
21	STAD	112	1177	2	0.02	0.00
22	THCA	233	7566	3	0.01	0.00
23	UCEC	2405	1510	13	0.01	0.01

Table S3: List of 28 DCSPs responding to anti-cancer drugs in the GDSC cohort and the BeatAML cohort.

	Pathway	Drug	t-statistics	cor-GDSC	cor-beatAML
1	MARTENS_BOUND_BY_PML_RARA_FUSION	Quizartinib	10.92	-0.14	-0.21
2	EPPERT_HSC_R	Quizartinib	10.28	-0.17	-0.21
3	TAKEDA_TARGETS_OF_NUP98_HOXA9_FUSION_6HR_UP	Quizartinib	10.25	-0.18	-0.19
4	BYSTRYKH_HEMATOPOIESIS_STEM_CELL_FLI1	Sorafenib	10.21	-0.30	-0.27
5	GAZDA_DIAMOND_BLACKFAN_ANEMIA_PROGENITOR_DN	Sunitinib	10.14	-0.24	-0.20
6	BYSTRYKH_HEMATOPOIESIS_STEM_CELL_FLI1	Cabozantinib	10.07	-0.27	-0.20
7	GAZDA_DIAMOND_BLACKFAN_ANEMIA_PROGENITOR_DN	Sorafenib	10.01	-0.27	-0.28
8	GAZDA_DIAMOND_BLACKFAN_ANEMIA_PROGENITOR_DN	Cabozantinib	9.73	-0.28	-0.20
9	BYSTRYKH_HEMATOPOIESIS_STEM_CELL_FLI1	Sunitinib	9.63	-0.27	-0.19
10	DIAZ_CHRONIC_MEYLOGENOUS_LEUKEMIA_DN	Quizartinib	9.10	-0.15	-0.22
11	BYSTRYKH_HEMATOPOIESIS_STEM_CELL_FLI1	Quizartinib	8.96	-0.17	-0.19
12	GAZDA_DIAMOND_BLACKFAN_ANEMIA_PROGENITOR_DN	Quizartinib	8.53	-0.16	-0.21
13	EPPERT_CE_HSC_LSC	Quizartinib	8.21	-0.16	-0.22
14	WAKABAYASHI_ADIPOGENESIS_PPARG_RXRA_BOUND_36HR	Nilotinib	7.60	-0.30	-0.27
15	RASHI_RESPONSE_TO_IONIZING_RADIATION_6	Palbociclib	7.16	-0.20	-0.33
16	WANG_IMMORTALIZED_BY_HOXA9_AND_MEIS1_DN	Quizartinib	6.79	-0.16	-0.21
17	NADLER_HYPERGLYCEMIA_AT_OBESITY	Quizartinib	6.35	-0.18	-0.22
18	JIANG_AGING_HYPOTHALAMUS_UP	Linifanib	5.98	-0.35	-0.31
19	NADLER_HYPERGLYCEMIA_AT_OBESITY	Cabozantinib	5.78	-0.42	-0.21
20	RASHI_RESPONSE_TO_IONIZING_RADIATION_6	Linifanib	4.94	-0.32	-0.30
21	KYNG_ENVIRONMENTAL_STRESS_RESPONSE_NOT_BY_4NQO_IN_OLD	Lestaurtinib	4.61	-0.25	-0.20
22	KRIEG_KDM3A_TARGETS_NOT_HYPOXIA	Selumetinib	4.34	-0.35	-0.19
23	RUAN_RESPONSE_TO_TROGLITAZONE_DN	Lenalidomide	4.18	-0.33	-0.29
24	INGRAM_SHH_TARGETS_DN	Lestaurtinib	3.70	-0.24	-0.20
25	WEI_MIR34A_TARGETS	Lenalidomide	3.56	-0.22	-0.20
26	RASHI_RESPONSE_TO_IONIZING_RADIATION_6	GW-2580	3.42	-0.37	-0.22
27	VALK_AML_WITH_T_8_21_TRANSLOCATION	Lestaurtinib	3.24	-0.16	-0.20
28	NADLER_OBESITY_UP	GW-2580	2.95	-0.34	-0.22

Table S4: PAS of pathway P_j treated by drug D_i in comparison with PASs of following three groups: 1) same pathway but different drugs ($\overline{D_i}$, P_j), 2) same drug but different pathways (D_i, $\overline{P_j}$), and 3) different drugs and different pathways ($\overline{D_i}$, $\overline{P_j}$). The values in the last four columns are median PAS of the groups. P-values of the permutation tests are reported in parentheses.

	**Pathway**	**Drug**	$\mathbf{D_i, P_j}$	$\mathbf{\overline{D_i}, P_j}$	$\mathbf{D_i, \overline{P_j}}$	$\mathbf{\overline{D_i}, \overline{P_j}}$
1	Quizartinib	MARTENS_BOUND_BY_PML_RARA_FUSION	24.34	0 (1e-4)	0 (1e-4)	0 (1e-4)
2	Quizartinib	EPPERT_HSC_R	25.02	0 (1e-4)	0 (1e-4)	0 (1e-4)
3	Quizartinib	TAKEDA_TARGETS_OF_NUP98_HOXA9_FUSION_6HR_UP	18.99	0 (1e-4)	0 (1e-4)	0 (1e-4)
4	Sorafenib	BYSTRYKH_HEMATOPOIESIS_STEM_CELL_FLI1	21.16	0 (1e-4)	10.66 (1)	0 (1e-4)
5	Sunitinib	GAZDA_DIAMOND_BLACKFAN_ANEMIA_PROGENITOR_DN	17.95	0 (1e-4)	0 (1e-4)	0 (1e-4)
6	Cabozantinib	BYSTRYKH_HEMATOPOIESIS_STEM_CELL_FLI1	21.26	0 (1e-4)	0 (1e-4)	0 (1e-4)
7	Sorafenib	GAZDA_DIAMOND_BLACKFAN_ANEMIA_PROGENITOR_DN	20.03	0 (1e-4)	10.66 (1)	0 (1e-4)
8	Cabozantinib	GAZDA_DIAMOND_BLACKFAN_ANEMIA_PROGENITOR_DN	20.42	0 (1e-4)	0 (1e-4)	0 (1e-4)
9	Sunitinib	BYSTRYKH_HEMATOPOIESIS_STEM_CELL_FLI1	21.6	0 (1e-4)	0 (1e-4)	0 (1e-4)
10	Quizartinib	DIAZ_CHRONIC_MEYLOGENOUS_LEUKEMIA_DN	22.76	0 (1e-4)	0 (1e-4)	0 (1e-4)
11	Quizartinib	BYSTRYKH_HEMATOPOIESIS_STEM_CELL_FLI1	20.35	0 (7e-3)	0 (1e-4)	0 (1e-4)
12	Quizartinib	GAZDA_DIAMOND_BLACKFAN_ANEMIA_PROGENITOR_DN	21.45	0 (1e-4)	0 (1e-4)	0 (1e-4)
13	Quizartinib	EPPERT_CE_HSC_LSC	25.76	0 (1e-4)	0 (1e-4)	0 (1e-4)
14	Nilotinib	WAKABAYASHI_ADIPOGENESIS_PPARG_RXRA_BOUND_36HR	1.84	0 (1e-4)	0 (1e-4)	0 (1e-4)
15	Palbociclib	RASHI_RESPONSE_TO_IONIZING_RADIATION_6	12.69	0 (1e-4)	0 (1e-4)	0 (1e-4)
16	Quizartinib	WANG_IMMORTALIZED_BY_HOXA9_AND_MEIS1_DN	18.94	0 (1e-4)	0 (1e-4)	0 (1e-4)
17	Quizartinib	NADLER_HYPERGLYCEMIA_AT_OBESITY	21.61	0 (1e-4)	0 (1e-4)	0 (1e-4)
18	Linifanib	JIANG_AGING_HYPOTHALAMUS_UP	7.25	0 (1e-4)	0 (1e-4)	0 (1e-4)
19	Cabozantinib	NADLER_HYPERGLYCEMIA_AT_OBESITY	25.57	0 (1e-4)	0 (1e-4)	0 (1e-4)
20	Linifanib	RASHI_RESPONSE_TO_IONIZING_RADIATION_6	10.03	0 (1e-4)	0 (1e-4)	0 (1e-4)
21	Lestaurtinib	KYNG_ENVIRONMENTAL_STRESS_RESPONSE_NOT_BY_4NQO_IN_OLD	11.74	0 (1e-4)	0 (1e-4)	0 (1e-4)
22	Selumetinib	KRIEG_KDM3A_TARGETS_NOT_HYPOXIA	3.21	0 (1e-4)	0 (1e-4)	0 (1e-4)
23	Lenalidomide	RUAN_RESPONSE_TO_TROGLITAZONE_DN	20.83	0 (1e-4)	0 (1e-4)	0 (1e-4)
24	Lestaurtinib	INGRAM_SHH_TARGETS_DN	11.72	0 (1e-4)	0 (1e-4)	0 (1e-4)
25	Lenalidomide	WEI_MIR34A_TARGETS	16.79	0 (1e-4)	0 (1e-4)	0 (1e-4)
26	GW-2580	RASHI_RESPONSE_TO_IONIZING_RADIATION_6	10.4	0 (1e-4)	0 (1e-4)	0 (1e-4)
27	Lestaurtinib	VALK_AML_WITH_T_8_21_TRANSLOCATION	11.53	0 (1e-4)	0 (1e-4)	0 (1e-4)
28	GW-2580	NADLER_OBESITY_UP	9.83	0 (1e-4)	0 (1e-4)	0 (1e-4)

FIGURES

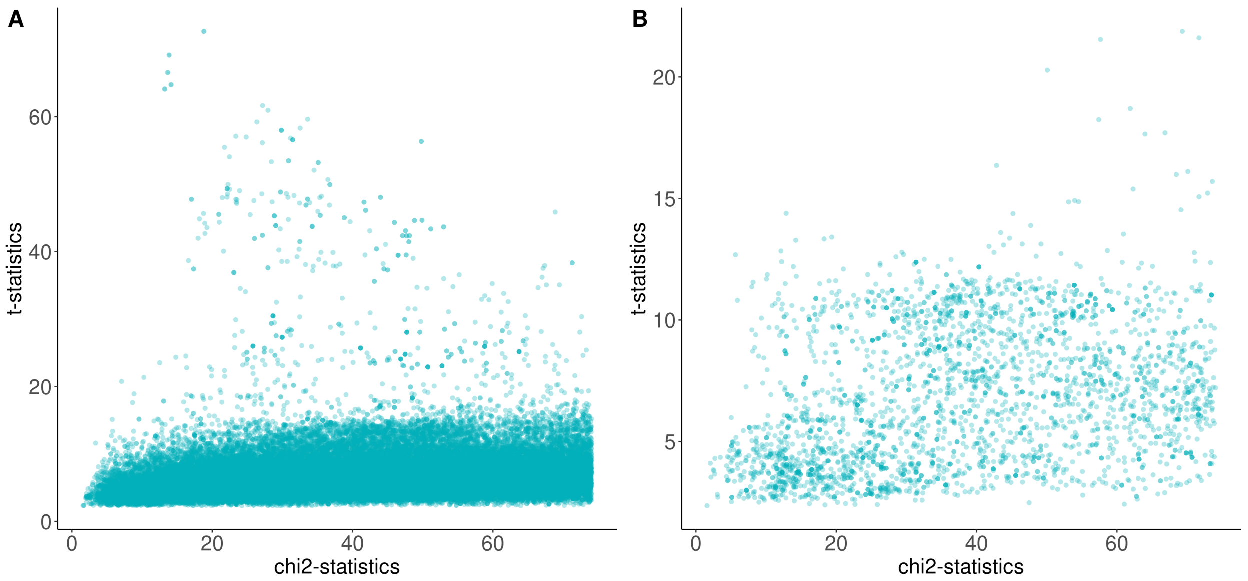

Figure S1: T-statistics and χ^2-statistics of DCSP candidates from the GDSC cohort: (A) Across 23 cancers and (B) Acute Myeloid Leukemia (AML).

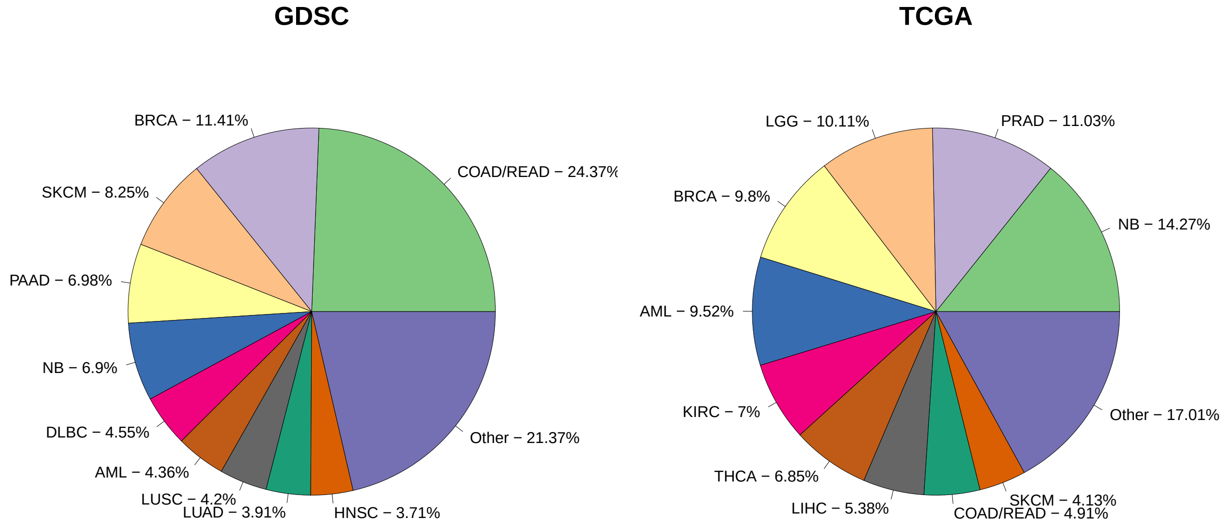

Figure S2: The proportion of identified DCSPs for individual diseases.

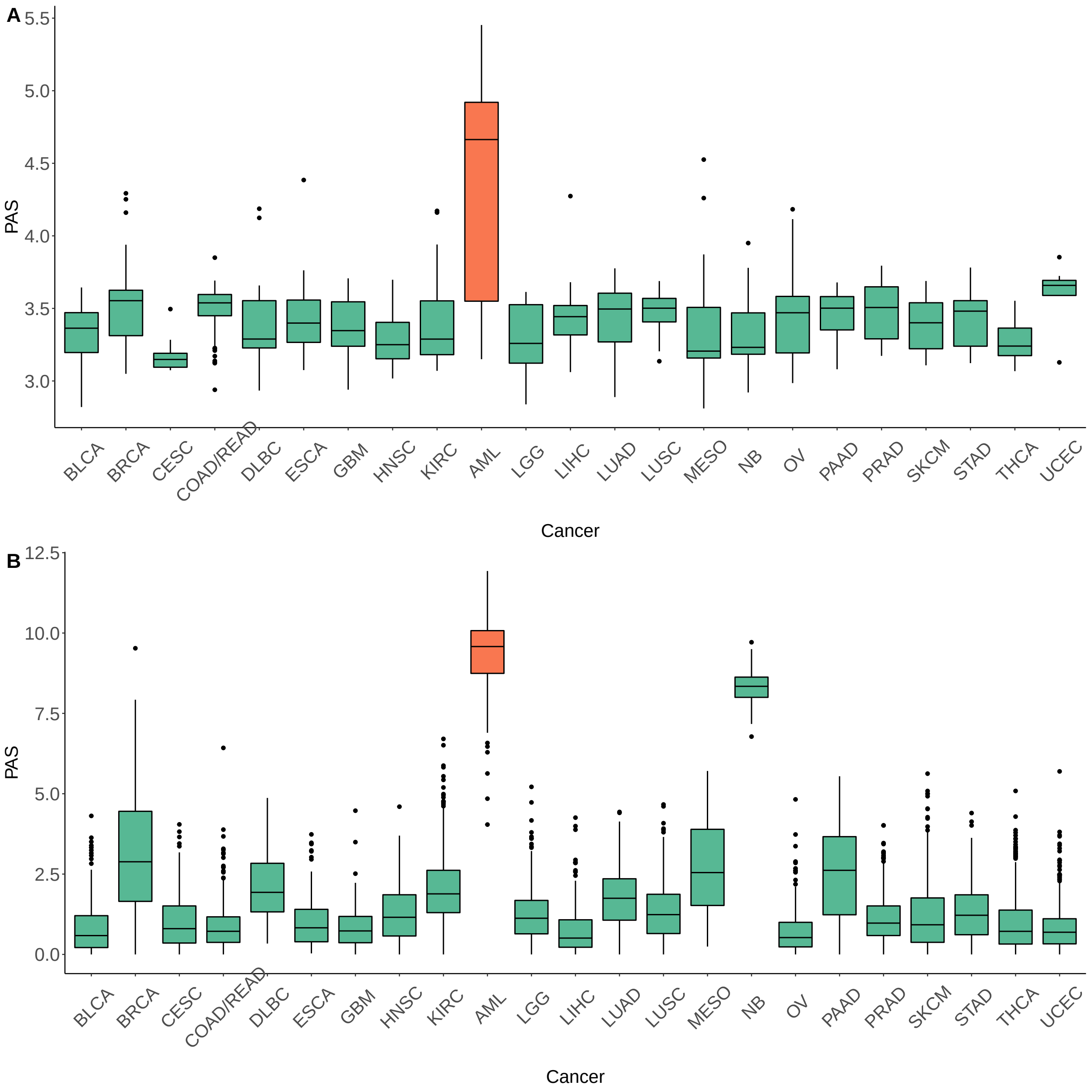

Figure S3: PASs of Martens-PML-RARA druggable by quizartinib in **A** - GDSC cohort, **B** - TCGA cohort. The orange box plots represent PAS of AML, and the green ones represent PASs of other cancers.

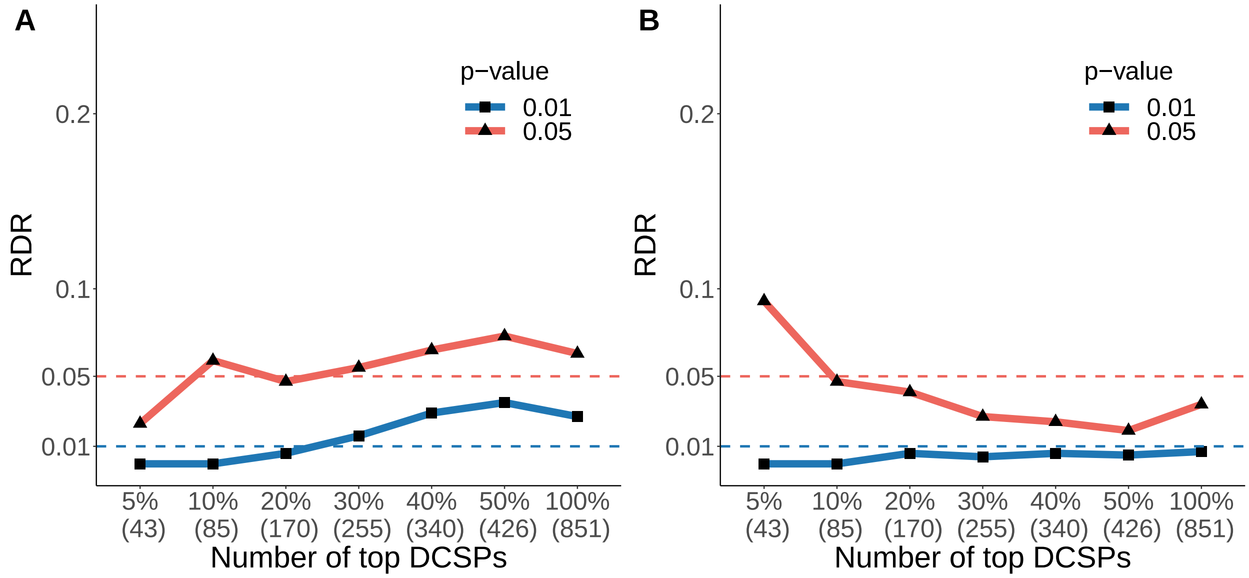

Figure S4: The rediscovery rate (RDR) of DCSPs in terms of the association between PAS and drug sensitivity in AML. RDR is the proportion of the top DCSPs identified in the GDSC cohort that is significant in the BeatAML cohort. Here, PAS is defined as PAS_d - PAS_u so a higher value of PAS indicates a higher activation of downstream part than of upstream part. (A) RDR of DCSPs with negative correlations and (C) RDR of DCSPs with positive correlations. The horizontal dashed lines present the p-value target lines (0.05 and 0.01). The RDRs follow the null target lines (0.05 and 0.01), i.e. null results, indicating that there is no evidence of drug response when the pathway has higher activation downstream rather upstream of the drug targets.
