# Supplementary material for: Discovery of druggable cancer-specific pathways with application in acute myeloid leukemia
Source: Gigascience. 2022 Sep 29;11:giac091. doi: 10.1093/gigascience/giac091 (PMC9520771; doi:10.1093/gigascience/giac091)
Supplement: giac091_Response_to_Reviewer_Comments_Original_Submission [file giac091_response_to_reviewer_comments_original_submission.pdf]

Author's Response To Reviewer Comments

Close

We have prepared point-by-point responses to specific reviewer and editor comments, however the responses contain some figures which are not able to included in this "Respond to Reviewers" box of the submission system. Therefore, we concatenated the response letter to the main text in a single file. Please see the details of the responses in the Manuscript file.

Close
