# Supplementary material for: Discovery of druggable cancer-specific pathways with application in acute myeloid leukemia
Source: Gigascience. 2022 Sep 29;11:giac091. doi: 10.1093/gigascience/giac091 (PMC9520771; doi:10.1093/gigascience/giac091)
Supplement: giac091_Response_to_Reviewer_Comments_Revision_1 [file giac091_response_to_reviewer_comments_revision_1.pdf]

Author's Response To Reviewer Comments

Close

For convenience, the letter of response to reviewers is concatenated with the main text in a single file

Close
