# Supplementary material for: Discovery of druggable cancer-specific pathways with application in acute myeloid leukemia
Source: Gigascience. 2022 Sep 29;11:giac091. doi: 10.1093/gigascience/giac091 (PMC9520771; doi:10.1093/gigascience/giac091)
Supplement: giac091_Response_to_Reviewer_Comments_Revision_2 [file giac091_response_to_reviewer_comments_revision_2.pdf]

# Author's Response To Reviewer Comments

Close

Dear editor,

All your comments have been addressed following the suggestions. The final version of in pdf format is provided in the upload system and the latex files are sent to you via email.

Thank you for your support.

Best wishes,
Nghia

Close
