# Supplementary material for: Discovery of druggable cancer-specific pathways with application in acute myeloid leukemia
Source: Gigascience. 2022 Sep 29;11:giac091. doi: 10.1093/gigascience/giac091 (PMC9520771; doi:10.1093/gigascience/giac091)
Supplement: giac091_Reviewer_1_Report_Original_Submission — Jing Tang -- 5/17/2022 Reviewed [file giac091_reviewer_1_report_original_submission.pdf]

Reviewer Report

Title: Discovery of Druggable Cancer-Specific Pathways with Application in Acute Myeloid Leukemia

Version: Original Submission **Date:** 5/17/2022

Reviewer name: Jing Tang

Reviewer Comments to Author:

Discovery of druggable cancer-specific pathways with application in AML
Authors developed a method to identify druggable cancer-specific pathways (CSPs). Pathway activity score (PAS) is measured by gene expressions and then weighted by the functional connectivity as well as relatedness to cancer driver genes and drug targets. Drugs that can target these pathways are found to be more sensitive, inferred from the GDSC cancer cell line data and then validated in the BeatAML cohort. The analysis involves an integration of multiple data types from multiple resources across cell lines to patient-derived samples. I think the topic fits the scope of the journal and the conclusion is generally valid. However, some major comments are given to improve the clarity.
Major comments:
1. Rationale of separating upstream versus downstream genes. Why "less or no response" of a drug is expected "if the driver gene is downstream of the target"? I would assume that the opposite is true.
2. The PAS is calculated as the product of the gene expression values and their weights. It seems to me that the PAS will favor highly expressed genes, which may not be directly correlated to cancer-specific or drug-specific pathways.
3. Difference between cancer-specific pathways or drug-specific pathways. It seems that the pathways are for a specific cancer-drug pair (e.g. Figure 1A), but how to predict drugs from these pathways?
4. Figure 1A and C are not consistent, as the average of PAS for AML is around 4.5 in Figure1A, but in Figure1C the average is around 0.
5. The 251,615 candidates are meant for candidate pathways?
6. Among the cancer types, colon cancer has the largest number of CSPs, does it imply that the drug sensitivity on colon cancers will be higher than the other cancer types? or it means that gene expressions of these CSPs are higher than in colon cancer than the other cancer types?
7. Figure 2D. the y-axis is not log2 scale as described in the figure caption.
8. What does Figure S1 tell by correlating the two statistics?
9. In Table S4, what are the other drugs that target the same pathways? Most of these drugs do not have a significant PAS compared to the putative drug. What might be the explanation for it?
10. Recent studies on GDSC integration with beatAML could be reviewed in the introduction or discussion, e.g. https://pubmed.ncbi.nlm.nih.gov/35440130/.

Methods

Are the methods appropriate to the aims of the study, are they well described, and are necessary controls included? Choose an item.

Conclusions

Are the conclusions adequately supported by the data shown? Choose an item.

Reporting Standards

Does the manuscript adhere to the journal's guidelines on minimum standards of reporting? Choose an item.

Choose an item.

Statistics

Are you able to assess all statistics in the manuscript, including the appropriateness of statistical tests used? Choose an item.

Quality of Written English

Please indicate the quality of language in the manuscript: Choose an item.

Declaration of Competing Interests

Please complete a declaration of competing interests, considering the following questions:

- Have you in the past five years received reimbursements, fees, funding, or salary from an organisation that may in any way gain or lose financially from the publication of this manuscript, either now or in the future?
- Do you hold any stocks or shares in an organisation that may in any way gain or lose financially from the publication of this manuscript, either now or in the future?
- Do you hold or are you currently applying for any patents relating to the content of the manuscript?
- Have you received reimbursements, fees, funding, or salary from an organization that holds or has applied for patents relating to the content of the manuscript?
- Do you have any other financial competing interests?
- Do you have any non-financial competing interests in relation to this paper?

If you can answer no to all of the above, write 'I declare that I have no competing interests' below. If your reply is yes to any, please give details below.

I declare that I have no competing interests

I agree to the open peer review policy of the journal. I understand that my name will be included on my report to the authors and, if the manuscript is accepted for publication, my named report including any attachments I upload will be posted on the website along with the authors' responses. I agree for my

report to be made available under an Open Access Creative Commons CC-BY license (http://creativecommons.org/licenses/by/4.0/). I understand that any comments which I do not wish to be included in my named report can be included as confidential comments to the editors, which will not be published.

Choose an item.

To further support our reviewers, we have joined with Publons, where you can gain additional credit to further highlight your hard work (see: https://publons.com/journal/530/gigascience). On publication of this paper, your review will be automatically added to Publons, you can then choose whether or not to claim your Publons credit. I understand this statement.

Yes Choose an item.
