# Supplementary material for: Discovery of druggable cancer-specific pathways with application in acute myeloid leukemia
Source: Gigascience. 2022 Sep 29;11:giac091. doi: 10.1093/gigascience/giac091 (PMC9520771; doi:10.1093/gigascience/giac091)
Supplement: giac091_Reviewer_2_Report_Original_Submission — Sebastian Vosberg -- 5/19/2022 Reviewed [file giac091_reviewer_2_report_original_submission.pdf]

Reviewer Report

Title: Discovery of Druggable Cancer-Specific Pathways with Application in Acute Myeloid Leukemia

Version: Original Submission **Date:** 5/19/2022

Reviewer name: Sebastian Vosberg

Reviewer Comments to Author:

Trac et al. provide a computational approach to identify cellular pathways that can be druggable using targeted treatment approaches. To this end, the authors compute "pathway activity scores" (PASs) for pathway/drug combinations, based on mRNA expression of pathway members across publicly available data sets and a priori information about the drug target to evaluate upstream and downstream gene expression individually. This apporach is further extended by incorporating cancer entities to identify "cancer specific pathways" (CSPs). Finally, the results are set in context with data from hig throughput drug sensitivity screens with a focus on data from acute myeloid leukemia (AML) samples.
Evaluating the gene expression of upstream and downstream pathway members with respect to druggable target genes is an interesting approach and can be informative in terms of treatment-associated pathway activity. Both the results and the approach itself are able to contribute to the field. Still, several issues need to be adressed in order to improve the quality of the manuscript:
Major issues:
(1) improve overall structure
- approach and workflow are unclear in the beginning of the manuscript
- describe individual steps in the beginning (Introduction), introduce specific terms accordingly
- precisely describe the concepts of "PAS" and "CSP" when introdcuing them
- clearly describe how "CSPs" are ranked when showing the results of RDR
- please describe more clearly (also in the beginning) why in particular AML associated pathways are considered
(2) improve readability/understanding of the results
- precisely describe what "PAS" refers to, i.e. PASs are computed per pathways/drug pair (as it is written in the Methods section)
- precisely describe what "CSP" refers to, i.e. CSPs are computed per pathways/drug/cancer triplet (as it is written in the Methods section)
- if CSP does not refer to a (druggagble) pathway being specific to a particular (single!) cancer entity, this should be renamed to avoid confusions
- in the text, several numbers are given without proper explanation where they are coming from (e.g. "251,615 candidates", "1051 CSPs")
(3) improve reproducibility:
- unclear how "target genes" are defined, provide a list of target genes per drug (i.e. in table S3)
- unclear how up-/downstream genes within gene sets (e.g. as defined within MSigDB) are defined, please describe
- unclear how results were filtered based on correlation between PAS/AUC, please provide cutoffs

- unclear what is meant by "For our definition of PAS, we do not expect positive correlation, so this part can be used as a negative control". How can that be used as a negative control? Please elaborate.
- unclear what is meant with "target lines", to which the authors refer in Fig 2B/C
(4) correct for inconsistencies/errors:
- labeling of Fig 1 is inconsistent. In the legend, no figure panels are described, while in the figure itself, some parts (still not all) are labelled with A/B/C/D. The left part (workflow) has no label, the "toy examples" do have. Please use consistent labeling throughout the figure, legend, and text
- labeling of Fig 2 is incomplete/misleading
- please provide information which data is shown in Fig 2D (GDSC?)
- please rephrase "CSPs of the same pathway", doesnt make sense. Will be obsolete if "CSP" is renamed (see comment above)
- Fig 2: displaying the numbers of CSPs per entity on a log scale is not appropriate and misleading! this should be given in absolute numbers. The authors may consider to describe the validation as relative numbers in an independent figure panel to improve readability.
Minor issues:
(5) please elaborate on the description/discussion of results:
- can the authors elaborate on the differences in total CSPs found in the GDSC and TCGA data sets, as there are substantial differences?
- have the authors elaborated on different mutations within target genes? The authors describe the FLT3 as "The FLT3 mutation is the one of the most common mutations in AML caused by the internal tandem duplication of FLT3", which is only parly true. There are additional mutations in the gene which occur frequently in AML, with different impact on the protein. This should be at least considered in the text.
- please elaborate on the result that the PAS of the (Di,Pj) group and the (not-Di,Pj) group is significantly not different (Fig 2D) in more detail. The authors state that "This can be due to Martens-PML-RARA pathway in AML patients is also linked to other anti-AML drugs, not only quizartinib", but this pathway is not listed in combination with another drug in table S3.
(6) the last part of the last abstract about the "Martens-PML-RARA" pathway, quizartinib and FLT3 should be moved to the Results section.
(7) please re-check the language, as there are several minor mistakes in wording and/or grammar. The authors may consider to have the manuscript read by a native speaker

Methods

Are the methods appropriate to the aims of the study, are they well described, and are necessary controls included? Choose an item.

Conclusions

Are the conclusions adequately supported by the data shown? Choose an item.

Reporting Standards

Does the manuscript adhere to the journal's guidelines on [minimum standards of reporting?]() Choose an item.

Choose an item.

Statistics

Are you able to assess all statistics in the manuscript, including the appropriateness of statistical tests used? Choose an item.

Quality of Written English

Please indicate the quality of language in the manuscript: Choose an item.

Declaration of Competing Interests

Please complete a declaration of competing interests, considering the following questions:

- Have you in the past five years received reimbursements, fees, funding, or salary from an organisation that may in any way gain or lose financially from the publication of this manuscript, either now or in the future?
- Do you hold any stocks or shares in an organisation that may in any way gain or lose financially from the publication of this manuscript, either now or in the future?
- Do you hold or are you currently applying for any patents relating to the content of the manuscript?
- Have you received reimbursements, fees, funding, or salary from an organization that holds or has applied for patents relating to the content of the manuscript?
- Do you have any other financial competing interests?
- Do you have any non-financial competing interests in relation to this paper?

If you can answer no to all of the above, write 'I declare that I have no competing interests' below. If your reply is yes to any, please give details below.

I declare that I have no competing interests

I agree to the open peer review policy of the journal. I understand that my name will be included on my report to the authors and, if the manuscript is accepted for publication, my named report including any attachments I upload will be posted on the website along with the authors' responses. I agree for my report to be made available under an Open Access Creative Commons CC-BY license (http://creativecommons.org/licenses/by/4.0/). I understand that any comments which I do not wish to be included in my named report can be included as confidential comments to the editors, which will not be published.

Choose an item.

To further support our reviewers, we have joined with Publons, where you can gain additional credit to further highlight your hard work (see: https://publons.com/journal/530/gigascience). On publication of this paper, your review will be automatically added to Publons, you can then choose whether or not to claim your Publons credit. I understand this statement.

Yes Choose an item.
