# Supplementary material for: Discovery of druggable cancer-specific pathways with application in acute myeloid leukemia
Source: Gigascience. 2022 Sep 29;11:giac091. doi: 10.1093/gigascience/giac091 (PMC9520771; doi:10.1093/gigascience/giac091)
Supplement: giac091_Reviewer_2_Report_Revision_1 — Sebastian Vosberg -- 7/29/2022 Reviewed [file giac091_reviewer_2_report_revision_1.pdf]

Reviewer Report

Title: Discovery of Druggable Cancer-Specific Pathways with Application in Acute Myeloid Leukemia

Version: Revision 1 **Date:** 7/29/2022

Reviewer name: Sebastian Vosberg

Reviewer Comments to Author:

The authors have substantially improved the manuscript and adressed almost all of the comments. There are only minor suggestions to the manuscript:
- page 2, end of Introduction: Please elaborate quickly what is the difference and/or the advantage of your study as compared to the study published by Jafara et al. This is necessary to reflect your work in the context of similar studies.
- page 2, first paragraph of the Results section: PAS_u has not been introduced when using it for the first time. Please do so.
- page 2, second paragraph of the Results section: "Then, intuitively, in this case an inhibitor will not have a chance to work." This is actually not true! Instead, the authors could state that this is part of their hypothesis, and/or introduce this suggestion as part of a simplified model, which is used for this study.
- page 3, second paragraph: again, it is unclear for the reader where the number of 250,479 results from. This is not the potential number of all possible CSPs across the GDSC cohort. Please describe where this number is coming from.
- page 3, third paragraph: similary, if 4,794 is the number of validated CSPs out of 250,479, please describe it in this way. If not, please describe where the number is coming from.
- page 3, fourth paragraph: "...this part can be used as negative control." This is again not true, the authors cannot use such an observation as negative control! The authors "do not expect positive correlation", but there is no proof, that this expectation is actually true! The authors could state that "we consider this as negative control in our study" or similar.
- page 4, last paragraph of the Discussion: again, if the authors cite the work by Jafari et al., they should reflect their results in a short (1-sentence) summary, otherwise it is totally unclear why this work is cited here.

Methods

Are the methods appropriate to the aims of the study, are they well described, and are necessary controls included? Choose an item.

Conclusions

Are the conclusions adequately supported by the data shown? Choose an item.

Reporting Standards

Does the manuscript adhere to the journal's guidelines on minimum standards of reporting? Choose an item.

Choose an item.

Statistics

Are you able to assess all statistics in the manuscript, including the appropriateness of statistical tests used? Choose an item.

Quality of Written English

Please indicate the quality of language in the manuscript: Choose an item.

Declaration of Competing Interests

Please complete a declaration of competing interests, considering the following questions:

- Have you in the past five years received reimbursements, fees, funding, or salary from an organisation that may in any way gain or lose financially from the publication of this manuscript, either now or in the future?
- Do you hold any stocks or shares in an organisation that may in any way gain or lose financially from the publication of this manuscript, either now or in the future?
- Do you hold or are you currently applying for any patents relating to the content of the manuscript?
- Have you received reimbursements, fees, funding, or salary from an organization that holds or has applied for patents relating to the content of the manuscript?
- Do you have any other financial competing interests?
- Do you have any non-financial competing interests in relation to this paper?

If you can answer no to all of the above, write 'I declare that I have no competing interests' below. If your reply is yes to any, please give details below.

I declare that I have no competing interests

I agree to the open peer review policy of the journal. I understand that my name will be included on my report to the authors and, if the manuscript is accepted for publication, my named report including any attachments I upload will be posted on the website along with the authors' responses. I agree for my report to be made available under an Open Access Creative Commons CC-BY license (http://creativecommons.org/licenses/by/4.0/). I understand that any comments which I do not wish to be included in my named report can be included as confidential comments to the editors, which will not be published.

Choose an item.

To further support our reviewers, we have joined with Publons, where you can gain additional credit to further highlight your hard work (see: https://publons.com/journal/530/gigascience). On publication of this paper, your review will be automatically added to Publons, you can then choose whether or not to claim your Publons credit. I understand this statement.

Yes Choose an item.
